# The Effect of Probiotics and Synbiotics on Risk Factors Associated with Cardiometabolic Diseases in Healthy People—A Systematic Review and Meta-Analysis with Meta-Regression of Randomized Controlled Trials

**DOI:** 10.3390/jcm9061788

**Published:** 2020-06-08

**Authors:** Karolina Skonieczna-Żydecka, Karolina Kaźmierczak-Siedlecka, Mariusz Kaczmarczyk, Joanna Śliwa-Dominiak, Dominika Maciejewska, Katarzyna Janda, Ewa Stachowska, Beata Łoniewska, Damian Malinowski, Krzysztof Borecki, Wojciech Marlicz, Igor Łoniewski

**Affiliations:** 1 Department of Human Nutrition and Metabolomics, Pomeranian Medical University in Szczecin, 71-460 Szczecin, Poland; karzyd@pum.edu.pl (K.S.-Ż.); dmaciejewska.pum@gmail.com (D.M.); katarzyna.janda@pum.edu.pl (K.J.); ewa.stachowska@pum.edu.pl (E.S.); k.borecki.pum@gmail.com (K.B.); 2 Department of Surgical Oncology, Medical University of Gdansk, Smoluchowskiego 17, 80-214 Gdańsk, Poland; leokadia@gumed.edu.pl; 3 Department of Clinical and Molecular Biochemistry, Pomeranian Medical University in Szczecin, 70-111 Szczecin, Poland; mariush@pum.edu.pl; 4 Sanprobi sp. z o.o. sp. k. Kurza Stopka 5/c, 70-535 Szczecin, Poland; joanna.sliwa@sanprobi.pl; 5 Department of Neonatal Diseases, Pomeranian Medical University in Szczecin, 70-111 Szczecin, Poland; beatal@pum.edu.pl; 6 Department of Pharmacology, Pomeranian Medical University in Szczecin, 70-111 Szczecin, Poland; damian.malinowski@pum.edu.pl; 7 Department of Gastroenterology, Pomeranian Medical University, 71-252 Szczecin, Poland

**Keywords:** probiotic, synbiotic, microbiota, cardiometabolic

## Abstract

We aimed to systematically review the effectiveness of probiotic/synbiotic formulations to counteract cardiometabolic risk (CMR) in healthy people not receiving adjunctive medication. The systematic search (PubMed/MEDLINE/Embase) until 1 August 2019 was performed for randomized controlled trials in >20 adult patients. Random-effect meta-analysis subgroup and meta-regression analysis of co-primary (haemoglobin A1c (HbA1C), glucose, insulin, body weight, waist circumference (WC), body mass index (BMI), cholesterol, low-density lipoproteins (LDL), high-density lipoproteins (HDL), triglycerides, and blood pressure) and secondary outcomes (uric acid, plasminogen activator inhibitor-1–PAI-1, fibrinogen, and any variable related to inflammation/endothelial dysfunction). We included 61 trials (5422 persons). The mean time of probiotic administration was 67.01 ± 38.72 days. Most of probiotic strains were of *Lactobacillus* and *Bifidobacterium* genera. The other strains were *Streptococci*, *Enterococci*, and *Pediococci*. The daily probiotic dose varied between 10^6^ and 10^10^ colony-forming units (CFU)/gram. Probiotics/synbiotics counteracted CMR factors (endpoint data on BMI: standardized mean difference (SMD) = −0.156, *p* = 0.006 and difference in means (DM) = −0.45, *p* = 0.00 and on WC: SMD = −0.147, *p* = 0.05 and DM = −1.21, *p* = 0.02; change scores on WC: SMD = −0.166, *p* = 0.04 and DM = −1.35, *p* = 0.03) in healthy persons. Overweight/obese healthy people might additionally benefit from reducing total cholesterol concentration (change scores on WC in overweight/obese: SMD: −0.178, *p* = 0.049). Poor quality of probiotic-related trials make systematic reviews and meta-analyses difficult to conduct and draw definite conclusions. “Gold standard” methodology in probiotic studies awaits further development.

## 1. Introduction

Cardiovascular diseases (CVD) are the most prevalent noncommunicable disorders, with cardiometabolic risk factors (CMRF) including obesity [1], abnormal lipid profile and hypertension [2], insulin resistance, and aberrant glycaemia [3], playing a role in the pathogenesis. Increased consumption of unhealthy, high-calorie foods combined with a sedentary lifestyle further contribute to their poor outcomes [4,5]. In healthy persons, modestly skewed metabolic parameters may stand for the early onset CMRF [2].

Metabolic malfunctions of diverse nature, with epigenetic, hormonal, and infectious factors, are involved in the pathogenesis [6,7]. Intestinal microbiota actively participating in metabolism is an important factor regulating body metabolism [8]. Microorganisms, primarily bacteria, inhabiting our digestive tract actively participate in the digestion of nutrients and, through its metabolites, can regulate not only energy recovery from food but also lipogenesis or fat formation [9]. The mechanisms by which the gut microbiota can contribute to the pathogenesis of metabolic disorders include the short chain fatty acids (SCFAs) biosynthesis to triglycerides and glucose as well as the phenomenon of endotoxemia leading to increased blood levels of liposaccharide (LPS), which aggravates the process of systemic inflammation [10]. Both LPS and LPS-related inflammation have been linked to metabolic diseases, e.g., diabetes and insulin resistance (IR) [11].

The microbiota communicates with the host via toll-like receptors, nuclear factor-ĸB, and mitogen-activated protein kinase [12], which were shown to improve serum and glucose lipid concentration, to reduce insulin resistance [13,14], and to induce hypocholesterolemic effects [13]. Also, the products of the metabolic activity of the microbiota-predominant SCFAs were shown to regulate various metabolic processes [15]. These molecules after binding to G-protein-coupled receptors make the secretion of peptide YY, which lowers gut motility and augments nutrient absorption [16]. Also, butyrate serves as a source of energy for intestinal cells and improves tissue sensitivity to insulin, counteracting the development of type 2 diabetes. Together with propionic acid, it can stimulate the production of satiety hormones. Of note, butyrate can also stimulate the formation of fat cells and the storage of fat droplets in these cells, presumably through increased glucose uptake or participation in lipid formation. On the other hand, it may also inhibit lipolysis, which, together with stimulating glucose uptake and triglyceride synthesis, makes it a potential therapeutic agent in the fight against hyperglycemia and hyperlipidemia [17].

Considering these facts, metabolic impairment is at least a consequence of gut microbiota alteration. The use of probiotics and synbiotics to counteract metabolic disturbances has been reported. Probiotics are “live microorganisms that, when administered in adequate amounts, confer a health benefit on the host”, which has been confirmed in properly controlled studies [18]. Synbiotics are combinations of probiotics and prebiotics. Prebiotics are substrates that are selectively utilized by host microorganisms conferring a health benefit, which must be scientifically documented [19].

A few meta-analyses evaluating the efficacy of probiotics and synbiotics in persons diagnosed with diabetes or hypertension have been published [20,21,22]. However, early-onset CMRF have never been meta-analysed and reported in the literature. Therefore, we conducted the first systematic review and meta-analysis in healthy individuals. We hypothesized that probiotics/synbiotics would be superior to placebo yet would result in greater improvement of some metabolic indices—possibly via microbiota and/or inflammatory as well as gut barrier related pathways as assessed by biochemical parameter alterations—with very few adverse effects. We included studies in which clinically healthy people including those with excess body weight, those who are overweight, and those who are obese.

## 2. Methods

### 2.1. Search Strategy and Inclusion Criteria

Two independent authors (K.S.Z. and K.B.) searched PubMed/MEDLINE/Embase from database inception until 1 August 2019 for randomized controlled trials (RCTs) comparing probiotics and synbiotics with placebo/no-intervention/physical activity/diet to counteract cardiometabolic malfunctions in healthy people with normal weight or moderate/high-risk obesity (i.e., not exceeding 40 kg/m^2^).

The following search string was used in PubMed (probiotic* OR synbiotic* OR microbiota* OR lactobacillus OR bifidobacterium) AND (RCT OR random* OR placebo*) AND (“hemoglobin A1C” OR HbA1C OR glucose OR “fasting glucose” OR “glucose tolerance” OR hyperglycemia OR “oral glucose tolerance test” OR OGTT insulin OR hyperinsulinemia OR “insulin resistance” OR IR OR “insulin sensitivity” OR weight OR obesity OR obese OR overweight OR over-weight OR weight-gain OR “waist circumference” OR “body mass index” OR BMI OR cholesterol OR LDL OR HDL OR triglycerides OR dyslipidemia OR lipid OR “blood pressure” OR SBP OR DBP OR uric acid OR “Plasminogen activator inhibitor-1” OR PAI-1 OR PAI1 OR fibrinogen OR inflamma* OR C-reactive OR “C-reactive protein” OR CRP OR WBC OR leukocytes OR lymphoctes OR endothel* OR “endothelial dysfunction”). In the Embase database, the search string was (‘normal human’/exp OR ‘healthy adult’ OR ‘healthy human’ OR ‘healthy humans’ OR ‘healthy patient’ OR ‘healthy people’ OR ‘healthy person’ OR ‘healthy subject’ OR ‘healthy subjects’ OR ‘healthy volunteer’ OR ‘healthy volunteers’ OR ‘human, normal’ OR ‘normal human’ OR ‘normal humans’ OR ‘normal subject’ OR ‘normal subjects’ OR ‘normal volunteer’ OR ‘normal volunteers’) AND (‘probiotic agent’/exp OR ‘probiotic’ OR ‘probiotic agent’ OR ‘probiotics’ OR ‘synbiotic agent’/exp OR ‘synbiotic’ OR ‘synbiotic agent’ OR ‘synbiotics’ OR ‘microflora’/exp OR ‘microbial flora’ OR ‘microbiota’ OR ‘microflora’ OR ‘lactobacillus’/exp OR ‘bifidobacterium’/exp) AND (‘glycosylated hemoglobin’/exp OR ‘glycated haemoglobin’ OR ‘glycated hemoglobin’ OR ‘glycated hemoglobin a’ OR ‘glycohaemoglobin’ OR ‘glycohemoglobin’ OR ‘glycosyl haemoglobin’ OR ‘glycosyl hemoglobin’ OR ‘glycosylated haemoglobin’ OR ‘glycosylated hemoglobin’ OR ‘glycosylhaemoglobin’ OR ‘glycosylhemoglobin’ OR ‘glycosylised haemoglobin’ OR ‘glycosylized hemoglobin’ OR ‘haemoglobin a1’ OR ‘haemoglobin a 1’ OR ‘haemoglobin a, glycosylated’ OR ‘haemoglobin ai’ OR ‘haemoglobin alpha 1’ OR ‘haemoglobin glycoside’ OR ‘haemoglobin glycosylation’ OR ‘hemoglobin a, glycosylated’ OR ‘hemoglobin glycoside’ OR ‘glucose’/exp OR ‘glucose’ OR ‘fasting blood glucose’/exp OR ‘fasting plasma glucose’/exp OR ‘insulin’/exp OR ‘insulin’ OR ‘insuline’ OR ‘insulin resistance’/exp OR ‘insulin resistance’ OR ‘resistance, insuline’ OR ‘insulin sensitivity’/exp OR ‘insulin insensitivity’ OR ‘insulin sensitivity’ OR ‘insulin sensitivity test’ OR ‘insulin test’ OR ‘sensitivity, insulin’ OR ‘hyperglycemia’/exp OR ‘glucose blood level, elevated’ OR ‘glycemia, hyper’ OR ‘hyperglycaemia’ OR ‘hyperglycemia’ OR ‘hyperglycemic syndrome’ OR ‘glucose tolerance test’/exp OR ‘gtt’ OR ‘g.t.t.’ OR ‘glucogram’ OR ‘glucose load’ OR ‘glucose loading test’ OR ‘glucose test’ OR ‘glucose tolerance curve’ OR ‘glucose tolerance factor’ OR ‘glucose tolerance test’ OR ‘glucose toleration test’ OR ‘body weight’/exp OR ‘body weight’ OR ‘total body weight’ OR ‘weight, body’ OR ‘waist circumference’/exp OR ‘waist circumference’ OR ‘waist size’ OR ‘body mass’/exp OR ‘bmi (body mass index)’ OR ‘quetelet index’ OR ‘body ban mass’ OR ‘body mass’ OR ‘body mass index’ OR ‘cholesterol’/exp OR ‘cholesterol’ OR ‘low density lipoprotein cholesterol’/exp OR ‘ldl cholesterol’ OR ‘cholesterol, ldl’ OR ‘lipoproteins, ldl cholesterol’ OR ‘low density lipoprotein cholesterol’ OR ‘high density lipoprotein cholesterol’/exp OR ‘hdl cholesterol’ OR ‘cholesterol, hdl’ OR ‘high density lipoprotein cholesterol’ OR ‘lipoproteins, hdl cholesterol’ OR ‘triacylglycerol’/exp OR ‘triacylglycerol’ OR ‘triglyceride’ OR ‘triglycerides’ OR ‘tryglyceride’ OR ‘dyslipidemia’/exp OR ‘dyslipaemia’ OR ‘dyslipemia’ OR ‘dyslipidaemia’ OR ‘dyslipidaemias’ OR ‘dyslipidemia’ OR ‘dyslipidemias’ OR ‘blood pressure’/exp OR ‘blood pressure’ OR ‘blood tension’ OR ‘pressure, blood’ OR ‘vascular pressure’ OR ‘plasminogen activator’/exp OR ‘fibrinogen’/exp OR ‘factor 1’ OR ‘factor i’ OR ‘fibrinogen’ OR ‘human fibrinogen’ OR, OR ‘inflammation’/exp OR ‘acute inflammation’ OR ‘bacterial inflammation’ OR ‘inflammation’ OR ‘inflammation reaction’ OR ‘inflammation response’ OR ‘inflammatory condition’ OR ‘inflammatory lesion’ OR ‘inflammatory process’ OR ‘inflammatory reaction’ OR ‘inflammatory response’ OR ‘inflammatory syndrome’ OR ‘reaction, inflammation’ OR ‘response, inflammatory’ OR ‘serositis’ OR ‘sterile inflammation’) AND (‘randomized controlled trial’/exp OR ‘controlled trial, randomized’ OR ‘randomised controlled study’ OR ‘randomised controlled trial’ OR ‘randomized controlled study’ OR ‘randomized controlled trial’ OR ‘trial, randomized controlled’).

A manual review of reference lists from the most recent reviews followed the electronic search. Inclusion criteria were (i) full-text randomized controlled trial, (ii) populations containing >20 adult (>18 years old participants, excluding pregnant women), (iii) treatment with pro-/synbiotics for at least 4 weeks, (iv) randomization to probiotic/synbiotic vs. controls (placebo, no intervention, physical activity, and dietary elements, e.g., yoghurts and milk), and (v) available meta-analyzable change score/endpoint data on any of the following outcomes: HbA1C OR glucose OR OGTT OR insulin OR weight OR waist circumference OR BMI OR cholesterol OR LDL OR HDL OR triglycerides OR blood pressure OR SBP OR DBP OR uric acid OR Plasminogen activator inhibitor-1 OR fibrinogen OR any outcome related to inflammation/endothelial dysfunction. The exclusion criteria were as follows: (i) intervention with microbial agent and adjunctive medication aiming or known to prevent or counteract metabolic dysregulation, e.g., metformin, and (ii) disease, excluding morbid and super obese persons. Data from more than 2-arm studies were abstracted separately for particular comparators; however, placebos were preferentially selected, and regarding dietary comparators, products contained no lactic acid bacteria (e.g., milk vs. yoghurt).

### 2.2. Data Abstraction

We used the standard data extraction sheet according to our previous studies [23,24,25]. Due to a high number of studies included into metaanalysis, the abstraction stage was done by 4 independent authors. The study list was divided into two parts, and each was abstracted by 2 authors (the 1st part by K.S.Z. and K.B. and the 2nd part by D.M. and J.Ś.-D.). We abstracted data on the study design, the persons enrolled, and the probiotic intervention characteristics in accordance with the Preferred Reporting Items for Systematic Reviews and Meta-Analyses (PRISMA). For evaluation of the risk of bias (ROB) [26], we incorporated The Cochrane Collaboration’s tool and reported the number of low-risk assessments [26]. This was done by one investigator (D.M.). If some data were missing or difficult to abstract (e.g., from figures) for the review, authors were contacted via email twice, one week apart. All inconsistencies were resolved by senior author (W.M. and I.Ł.) consensus. Data from figures was extracted by means of WebPlotDigitizer software (https://automeris.io/WebPlotDigitizer/).

### 2.3. Outcomes

Co-primary outcomes were the changes within glycosylated haemoglobin A1c (HbA1C), glucose, insulin, Homeostatic Model Assessment of Insulin Resistance (HOMA-IR), body weight, waist circumference (WC), body mass index (BMI), lipid profile (total cholesterol, low-density lipoproteins (LDL), high-density lipoproteins (HDL), and triglycerides), and blood pressure. Secondary outcomes included uric acid, plasminogen activator inhibitor-1, fibrinogen, and any outcome related to inflammation/endothelial dysfunction (e.g., C-reactive protein (CRP) and leukocyte count). Additionally, we abstracted all-cause and adverse-events discontinuation.

### 2.4. Data Synthesis and Statistical Analysis

We conducted a random-effects [27] meta-analysis of outcomes for which ≥3 studies contributed data, using Comprehensive Meta-Analysis V3 (http://www.meta-analysis.com). We explored study heterogeneity using the chi-square test of homogeneity, with *p* < 0.05 indicating significant heterogeneity. All analyses were two-tailed with alpha = 0.05.

Group differences in continuous outcomes were analysed as the pooled standardized mean difference (SMD) in either endpoint scores (preferred) or change scores from endpoint to baseline (if endpoint scores were not available) using observed cases (OC). For continuous metabolic outcomes, standardized mean difference (SMD) and, where applicable, differences in means (DM) were calculated. The additional analyses included studies with participants with proper BMI value (20–25 kg/m^2^) and trials including overweight and obese persons (BMI > 25 kg/m^2^, not exceeding 45 kg/m^2^)

To understand the relationship between effect sizes and various study-level predictors, we fit random-effect meta-regression (multiple) models without interaction term using DerSimonian–Laird estimator estimation of the amount of heterogeneity. The test statistics of the individual coefficient (and confidence intervals) for predictors were based on standard normal distribution (z), and the overall test was based on the chi-square distribution (Q statistics following the chi-square distribution with degrees of freedom representing the number of predictors). Meta-regression variables included (i) number of low ROB assessments, (ii) study duration, (iii) mono- vs. multi-strain probiotic intervention, (iv) sample size (analysed persons), and (v) age of participants (mean). Finally, we evaluated funnel plots and conducted Egger’s regression test [28] to detect whether publication bias could have influenced the results we obtained.

## 3. Results

### 3.1. Search Results

**Figure 1:**
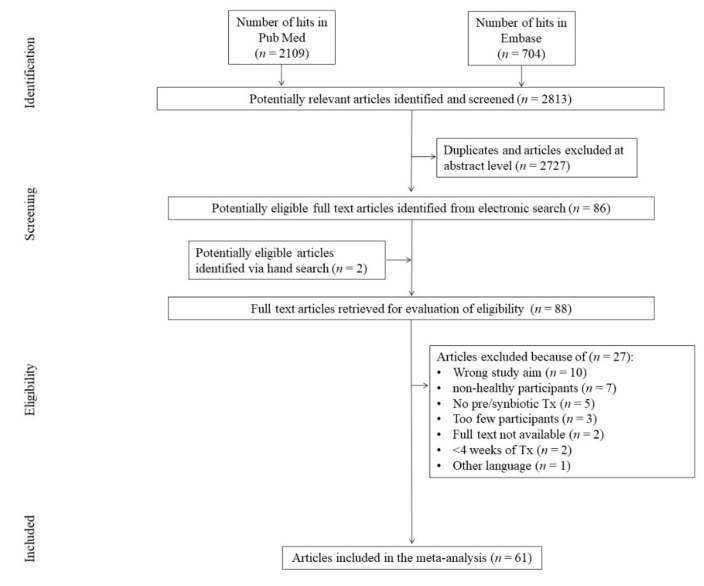
Study flow chart. Tx—treatment.

The initial search yielded 2813 hits. Almost 97% (*n* = 2727) of screened studies were excluded, being duplicates and/or after evaluation on the title/abstract level. Two (*n* = 2) additional articles were identified via hand search. After exclusion of duplicates between the initial search and hand search results, 88 (*n* = 88) full-text articles were reviewed. Of those, a total of 27 (*n* = 27) papers were excluded due to not fitting the inclusion criteria. The primary reasons for exclusion were wrong study aim (*n* = 10); non-healthy participants (*n* = 7); no probiotic treatment (*n* = 5); too few participants (*n* = 3); too short a study duration (*n* = 2); unavailability of full texts (*n* = 2); and another language other than English, German, and Polish (*n* = 1), yielding 61 (*n* = 61) studies that were included in the meta-analysis (Figure 1).

### 3.2. Study, Treatment, and Patient Characteristics

As demonstrated in Table 1, altogether, 61 studies (*n* = 61) were included [29,30,31,32,33,34,35,36,37,38,39,40,41,42,43,44,45,46,47,48,49,50,51,52,53,54,55,56,57,58,59,60,61,62,63,64,65,66,67,68,69,70,71,72,73,74,75,76,77,78,79,80,81,82,83,84,85,86,87,88,89], comprising 84 interventions. The mean probiotic administration was 67.01 ± 38.72 days (range = 28–186 days). Probiotic, not synbiotic, interventions were predominantly conducted (*n* = 54) [29,30,31,32,33,34,35,36,39,40,41,42,43,44,46,47,49,50,51,52,53,54,55,56,57,58,59,60,61,62,63,64,66,67,68,69,71,72,73,74,75,76,77,78,79,80,81,82,83,84,85,86,88,89]. Probiotic powders were administered in 15 studies [31,36,39,40,42,44,48,49,50,51,60,72,79,80,81], and in the cases of 14 [30,38,41,53,54,61,63,66,69,74,76,83,88,89] and 9 trials [29,33,43,52,56,57,58,84,86], yoghurt and milk products served as probiotic carriers, respectively. Almost all but eight of probiotic strains utilized in the trails belonged to *Lactobacillus* and *Bifidobacterium* genera. The other strains ingested by study participants were from *Streptococcus*, *Enterococcus*, and *Pediococcus* genera. The daily doses varied between 10^6^ and 10^10^ CFU (colony-forming units). The trials were financed by only industry budgets in 20 (*n* = 20) [29,30,33,34,39,41,42,46,47,48,55,64,67,69,75,76,78,79,83,84]. Studies were financed only by academic resources in 10 studies (*n* = 10) [31,32,35,37,40,43,44,52,58,60]. The sponsorships in other studies were partially academic/industrial/government.

All studies included healthy subjects (including overweight and obese but excluding morbidly obese persons), with a total of 6820 subjected to randomization and 5422 subjected to analysis. The overall mean age was 44.26 ± 12.87 (range: 21.43–71.9) years. The majority of studied persons were females (*n* = 2934, 57.22%). Baseline metabolic parameters of included persons are presented in Table S1, and the smoking status and diet along with physical activity are in Table S2. When analysing discontinuation events being consequences of adverse events, we found that the probiotic intervention was linked to very few adverse effects, the majority of which were of gastrointestinal origin. Apart from the most common bowel discomforts, i.e., nausea, diarrhea, constipation, and flatulence, there were also cardiac-related events, dental infections, chest tightness, sleep dysregulation, as well as hives. The details on are presented in Supplementary Table S3).

### 3.3. Risk of Bias Assessment

As evaluated by means of a ROB assessment tool, the mean number of low risks of bias assessment was 3 (median 2.5). The highest score, i.e., 7 low ROB assessments was detected in only one study [35] and 6 low ROB assessments were detected in two studies only [73,82]. Additionally, while analysing the papers, we detected a number of unclear risks of bias. The exact ROB evaluation in particular domains is in Table S4.

### 3.4. Effects on Metabolic Indices

**Figure 2:**
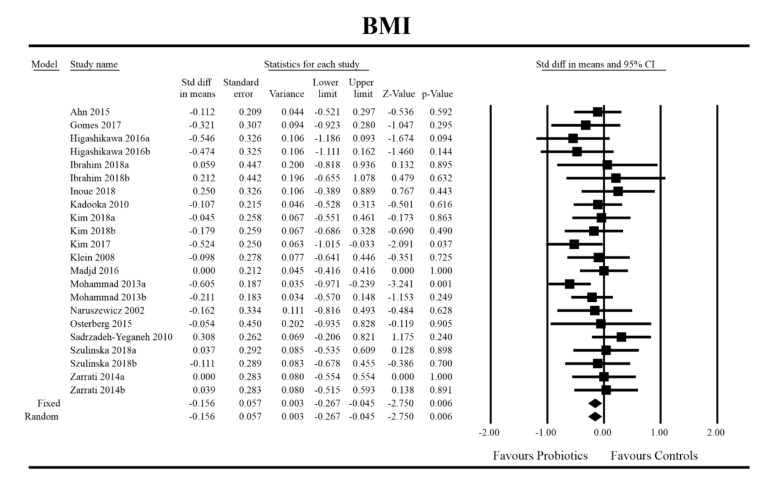
Effect size and standardized mean difference for BMI in persons taking probiotics vs. controls (endpoint data). Q = 18.487, df (Q) = 21, *p* = 0.618, I-squared = 0.0. BMI—body mass index. [31,44,49,50,51,56,59,60,61,66,67,68,72,74,81,89].

**Figure 3:**
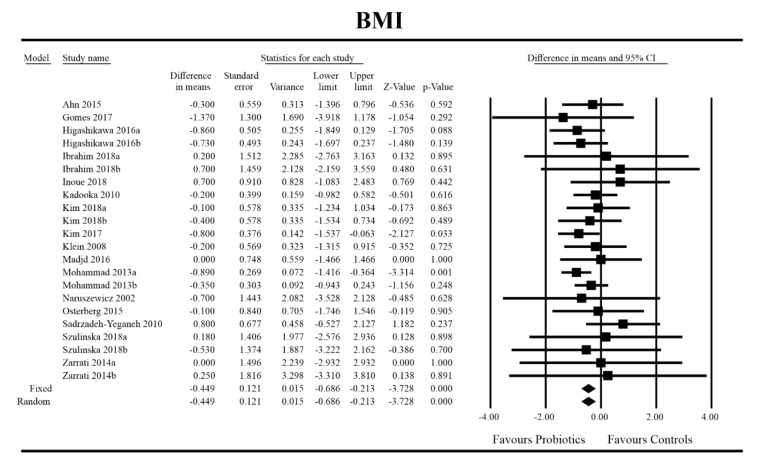
Effect size and difference in means for BMI in persons taking probiotics vs. controls (endpoint data). Q = 12.996, df (Q) = 21, *p* = 0.909, I-squared = 0.0. BMI—body mass index. [31,44,49,50,51,56,59,60,61,66,67,68,72,74,81,89].

**Figure 4:**
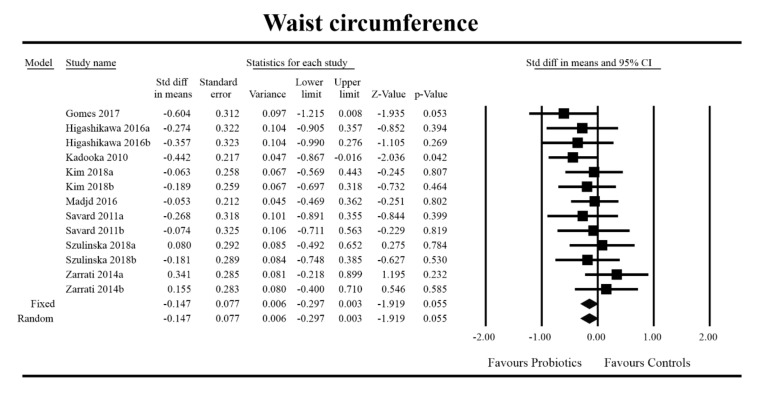
Effect size and standardized mean difference for waist circumference (WC) in persons taking probiotics vs. controls (endpoint data). Q = 9.773, df (Q) = 12, *p* = 0.636, I-squared = 0.0. WC—waist circumference. [44,49,56,59,66,76,80,81].

**Figure 5:**
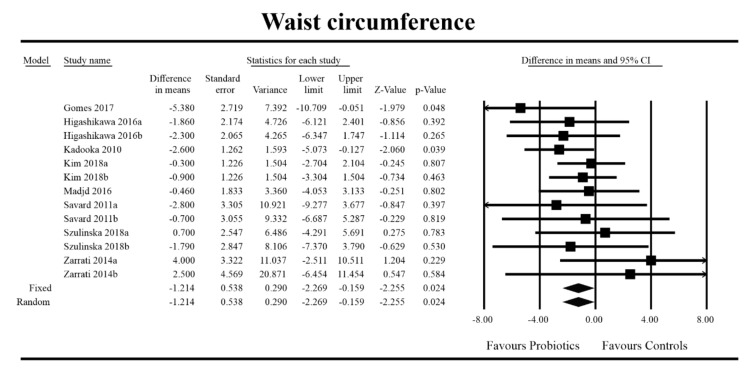
Effect size and difference in means for WC in persons taking probiotics vs. controls (endpoint data). Q = 8.698, df (Q) = 12, *p* = 0.729, I-squared = 0.0. WC—waist circumference. [44,49,56,59,66,76, 80,81].

**Figure 6:**
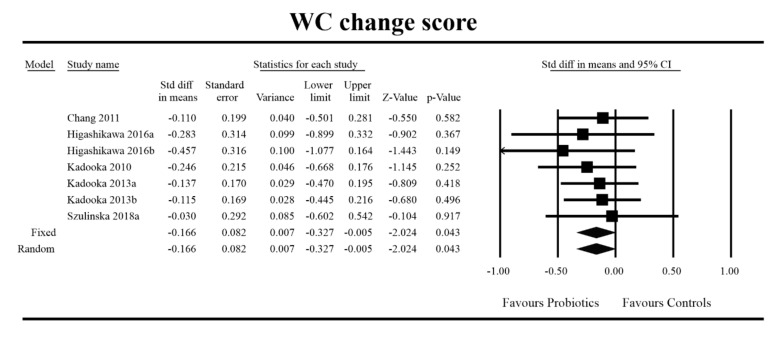
Effect size and standardized mean difference for WC in persons taking probiotics vs. controls (change scores). Q = 1.539, df (Q) = 6, *p* = 0.959, I-squared = 0.0. WC—waist circumference. [38,49,56,57,81].

**Figure 7:**
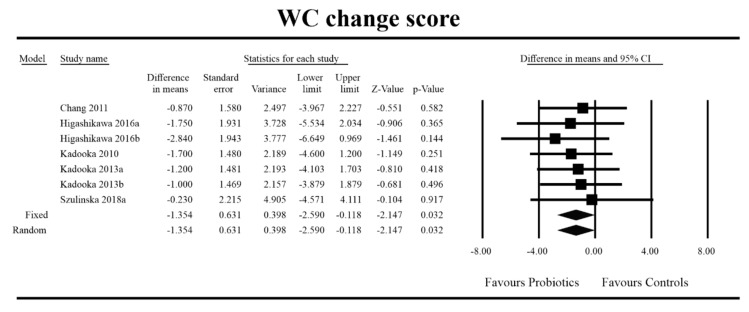
Effect size and difference in means for WC in persons taking probiotics vs. controls (change scores). Q = 1.102, df (Q) = 6 *p* = 0.981, I-squared = 0.0. WC—waist circumference. [38,49,56,57,81].

Out of all the metabolic indices that we evaluated, BMI and waist circumference decrease were significantly lower with the probiotic compared to controls. For endpoint data, the results were as follows: BMI studies = 16, *n* = 1256, SMD = −0.156, 95%CI = −0.27 to −0.04, *p* = 0.006 and DM = −0.45, 95%CI = −0.69 to −0.21, *p* = 0.00 and WC studies = 8, *n* = 690, SMD = −0.147, 95%CI = −0.30 to 0.03, *p* = 0.05 and DM = −1.21, 95%CI = −2.27 to −0.16, *p* = 0.02. In the case of the meta-analysis using change scores, the following results were obtained: WC studies = 5, *n* = 711, SMD = −0.166, 95%CI = −0.32 to −0.005, *p* = 0.04 and DM = −1.35, 95%CI = −2.59 to −2.15, *p* = 0.03 (Figure 2, Figure 3, Figure 4, Figure 5, Figure 6 and Figure 7). In one case, Egger’s test did indicate publication bias (DM for BMI: t value = 2.37, *p* = 0.02). For complete results, see Figures S1–S6.

As for the other metabolic indices, we found that probiotic ingestion in clinically healthy subjects did not affect those (Table S5).

### 3.5. Effects on Metabolic Indices Regarding Obesity Status

When conducting analysis by BMI status, i.e., in persons with BMI within normal (BMI: 20–25 kg/m^2^) and abnormal (BMI: >25 kg/m^2^) range, we were able to demonstrate that probiotic intake significantly affected total cholesterol (endpoint analyses) in persons with normal BMI value (SMD: −0.974; 95% CI: −1.661 to −0.286, *p* = 0.006). However, Egger’s test did indicate publication bias (SMD for total cholesterol (endpoint data): t value = 5.38, *p* = 0.000006). On the other hand, the analyses on the same parameter but regarding change scores depicted that probiotics significantly lowered the parameter in persons with abnormal BMI only (SMD: −0.206, 95% CI: −0.395 to −0.018, *p* = 0.032). In this case, no publication bias was detected (SMD for total cholesterol (change sores): t value = 1.64, *p* = 0.137). At last, we evaluated that WC (change score) was significant also in persons with abnormal BMI (SMD: −0.178, 95% CI: −0.354 to −0.001, *p* = 0.049). In this case, no publication bias was detected (SMD for total cholesterol (change sores): t value = 1.29, *p* = 0.265).

### 3.6. Metaregression Analyses

For endpoint data (SMD) regarding diastolic blood pressure (DBP), *p* values for all predictors were significant (Q = 19.22, df = 7, *p* = 0.0075): ROB (−0.31, z = −2.18, *p* = 0.029) and age (0.03, z = 2.07, *p* = 0.038), indicating that the predicted effect size decreases with increasing risk of bias and increases with age. The model explained 96% of the heterogeneity; however, the permutation test did not confirm the significance of predictors (*p* = 0.103 and *p* = 0.093, for ROB and age, respectively). In the case of insulin, *p* values for all predictors were found to be significant (Q = 16.37, df = 6, *p* = 0.012): number of low ROB assessments (−0.59, z = −2.57, *p* = 0.010), number of persons analysed (−0.04, z = −2.43, *p* = 0.015), duration of probiotic intervention (0.02, z = 2.27, *p* = 0.023), and BMI of analysed subjects (0.37, z = 3.15, *p* = 0.0016), indicating that the predicted effect size tended to decrease with ROB and study sample size, whereas with increasing duration and BMI, the effect size tended to be greater. The model did not explain heterogeneity, and the permutation test was nonsignificant. When analysing the triglycerides level, p values for all predictors were also significant (Q = 19.76, df = 7, *p* = 0.0061): monostrain vs. multistrain probiotics (−0.28, z = −2.47, *p* = 0.014) and BMI (0.056, z = 3.00, *p* = 0.003), indicating that the predicted effect size tended to be smaller for multistrain formulas whereas the effect size increased with increasing BMI. The model explained 90% of heterogeneity, and permutation tests were significant (*p* = 0.022 and *p* = 0.005 for type of formula and BMI, respectively). Finally, for total cholesterol, *p* values for all predictor were significant (Q = 90.55, df = 7, *p* < 0.0001): number of low ROB assessments (−0.67, z = −4.67, *p* < 0.0001), number of persons analysed (−0.06, z = −7.01, *p* < 0.0001), duration of probiotic intervention (0.02, z = 3.69, *p* = 0.0002), age (0.04, z = 2.60, *p* = 0.009), and BMI of participants (0.19, z = 2.98, *p* = 0.003). For BMI, HOMA-IR, LDL, and systolic blood pressure (SBP), *p* values for all predictors and for the effect sizes calculated for change score data were found to be nonsignificant (Q).

### 3.7. Microbiota Parameters

In 18 studies [35,36,40,48,55,61,63,65,72,75,76,77,79,81,82,83,85,90], microbiota and gut-barrier-related outcomes were evaluated following probiotic intervention. Composition and/or metabolites and/or immunological and/or gut-barrier-related outcomes were evaluated following probiotic intervention. These were data on faecal microbiota composition (*n* = 13), bacterial metabolites analyses (*n* = 9), as well as gut barrier integrity markers (mostly LPS, CRP, and zonulin) (*n* = 13) and various blood immune markers (mostly cytokines) (*n* = 12). Table 2 presents major results on these parameters. In the analysed studies, particular genera abundance was reported. Only in four studies, microbiota by means of next generation sequencing (NGS) technique was evaluated. Other trials utilized the culture-dependent technique and quantitative polymerase chain reaction (qPCR). We analysed also the association between clinical outcome, microbiota changes, anti-inflammatory effects, and gut barrier markers caused by probiotics administration (Supplementary Table S6). Clinical outcome was associated, in six studies, with microbial changes; in two studies, with microbial metabolites changes; and in two studies, with anti-inflammatory or gut barrier markers alterations. In four studies, changes of microbiota were observed despite lack of clinical efficacy of probiotic treatment. 

## 4. Discussion

In past years, many studies revealed that probiotics and synbiotics, through interactions with hosts, could affect nutrient metabolism and energy balance. Our current meta-analysis of 61 clinical trials and 5422 persons exclusively investigated the impact of probiotic and synbiotic interventions to reduce cardiovascular risk factors in otherwise healthy adults. The only factor we decided not to exclude was overweight and obesity, as their prevalence is worldwide and as they impact human’s health [91]. Morbidly obese persons (BMI ≥ 45 kg/m^2^) were excluded from the present analysis. We also decided to exclude studies with adjunct medications with reported efficacy against metabolic dysregulation (e.g., metformin [92]). Similarly, we excluded patients with diagnosed diseases, as meta-analyses in such patients have already been published [93,94,95] The results of the present meta-analysis indicated that probiotics may reduce the BMI by 0.5 unit (provide stats) and decrease waist circumference by more than 1.5 cm (stats). The effect sizes were/were not affected by meta-regression statistics. The up-to-date published data indicate that probiotics may reduce body weight, BMI, and other anthropometric indices, e.g., fat mass and waist circumference, via several mechanisms. While restoring the microecological ecosystem, probiotics diminish the inflammation responsible for insulin sensibility in the hypothalamus [96]. This in turn, together with increased concentration of glucagon-like peptide-1 (GLP-1) as well as peptide YY (PYY), improve satiety and suppress appetite by delaying gastric emptying. It should be emphasized that gut-derived GLP-1 is able to attenuate gut motility and to facilitate the aggregation of the constitutive flora to ferment more polysaccharides [97]. Furthermore, healthy microbiomes within the gut upregulate the expression of fasting-induced adipocyte factor (FIAF) and thus limits the degradation of lipoproteins and the deposition of free fatty acids in adipose tissue. Together with reduced food intake, the abovementioned healthy microbiome can promote reduction of body weight [96,98]. The systematic review by Crovesy et al. [96] indicated that strains of *Lactobacillus gasseri* and *Lactobacillus amylovorus* may promote decrease of body weight in the overweight population. The meta-analysis by John et al. [97] confirmed that probiotic therapy was associated with a significant reduction of BMI and, thus, body weight and fat mass. The study group consisted of overweight and obese persons. Notwithstanding, another systematic review and meta-analysis in a similar group of subjects showed that administration of probiotics was related to reduction of body weight in comparison to the placebo; however, the effect sizes were small (weighted mean difference (95% confidence interval); −0.60 (−1.19, −0.01) kg, I^2^ = 49%), BMI (−0.27 (−0.45, −0.08) kg m^−2^, I^2^ = 57%) and fat percentage (−0.60 (1.20, −0.01) %, I^2^ = 19%). Similarly to our findings, the effect of probiotics on fat mass was not significant (−0.42 (−1.08, 0.23) kg, I^2^ = 84%) [99]. Also, a study by Depommier et al. [100] demonstrated that supplementation with Akkermansia Muciniphila in overweight and obese human volunteers improved insulin sensitivity and total plasma cholesterol with a small reduction of body mass compared to controls. In contrast, in healthy, but overweight subjects, the administration of *Lactobacillus amylovorus* and *Lactobacillus fermentum* strains reduced this body fat mass [101].

The current meta-analysis did not confirm the efficacy of probiotics administration in reduction of other cardiovascular risk in healthy people. Of note, carbohydrate and lipid metabolism was not significantly affected by this type of intervention. In contrary to diabetic patients, we did not find any effect of probiotic therapy on carbohydrate metabolism. A study by Raygan et al. [102] which was conducted in patients with type 2 diabetes mellitus (T2DM) and coronary heart disease found that the intervention, during which the strains of *Bifidobacterium bifidum*, *Lactobacillus casei*, and *Lactobacillus acidophilus* were ingested for 12 weeks, significantly decreased the plasma glucose and insulin resistance. In a meta-analysis by Samah et al., [103] moderately hypoglicaemic properties (lower levels of fasting blood glucose) of microbial agents were confirmed. As in previously quoted studies, the meta-analysis cohort coincided with T2DM patients. Probiotics were demonstrated to affect glucose metabolism via several mechanisms, including antioxidant activity, and thus diminished gut-barrier integrity disruption, enhanced NK cells activity in the liver cells, and diminished insulin resistance by modulating the expression of proinflammatory cytokines and NF-kB-binding activity. Indeed, eubiosis within the gut may serve as a protective point for the preDM and DM onsets, diminishing low-grade inflammation which characterizes all metabolic diseases [104,105]. As concerns inflammation status, we did not find the relationship between common inflammatory markers (CRP and leukocytes count) as well as other indices associated with insulin resistance, including endothelial markers and uric acid. In T2DM patients, probiotics were found to lower the concentrations of hs-CRP, IL-6, and TNF-α [106]. Similar results, regarding hs-CRP, were demonstrated lately in a meta-analysis by Zheng et al. [107] and by Tabrizi et al. [108]. At last, the increase of the bioavailability of gliclazide regulating the intestinal absorption of glucose may also play a role [93].

In our study, we found that probiotics can decrease the total cholesterol level in persons with increased BMI, but other lipid parameters were not affected by probiotics and synbiotics administration. In Wang et al.’s meta-analysis including 32 randomized controlled trials (1971 participants with various metabolic entities), it was proved that probiotics significantly reduced serum total cholesterol (MD = −13.27, 95% CI (−16.74–9.80), *p* < 0.05) in comparison to controls [109]. Similar results were obtained in the meta-analyses by Chao et al. [110] and Shimizu et al. [111] (30 RCTs and 33 RCTs, respectively; hypocholesterolemic effects of probiotics–mean net change of total cholesterol: 7.8 mg/dL and 6.57 mg/dL, respectively, both in persons with mild lipid malfunctions). There are many hypotheses regarding mechanisms in which probiotics may lower the cholesterol level, such as binding of cholesterol to the probiotic cellular surface and incorporation of cholesterol molecules into the probiotic cellular membrane. However, the deconjugation of bile via bile salt hydrolase (BSH) activity seems to be the most profound mechanism in which probiotics reduce cholesterol level [112]. Bile salt hydrolase is the enzyme that catalyses the hydrolysis of glycine- and/or taurine-conjugated bile salts into amino acids residues and free bile acids. The most BSH-active probiotics belong to the genera of *Lactobacillus*, *Lactococcus*, and *Bifidobacterium*. These probiotics increase the production of bile salts from cholesterol in their colonized area and, as a consequence, contribute to reduced risk of coronary heart diseases [112].

The administration of probiotics improved blood pressure in humans, which was confirmed in Khalesi et al.’s meta-analysis including 9 randomized, controlled trials [113]. The consumption of probiotics significantly decreased systolic blood pressure by 3.56 mmHg and diastolic blood pressure by 2.38 mmHg in comparison to control groups (the duration of intervention is ≥8 weeks or daily dose > 10^11^ CFU). In contrast to our study, the authors included studies evaluating people with metabolic syndrome, hypertension, and hypercholesterolemia. As the menopause period is a strong contributor of CVD [114], we looked for metabolic effects on probiotic intake in this particular subgroup of participants. We were able to demonstrate that probiotic intake decreased the vascular stiffness in obese postmenopausal women [80]. Also, as reported by Lambert et al. [62], probiotics significantly diminished vasomotor symptoms of menopause. In a study by Szulińska et al. [81] was found that probiotics administration favorably affected the risk factors in a dose-dependent manner, showing beneficial effects on the cardiometabolic parameters and gut permeability of obese postmenopausal women. However, Brahe et al. [36] did not record that metabolic index was affected by microbial agent administration. Only these three studies reported on metabolic effects in the perimenopausal period; thus, we did not conduct a subgroup analysis. More studies are needed to clarify if and how probiotics can affect CVD risk in women at the menopause period.

Last but not least, we abstracted data related to the influence of probiotic administration on gut microbiota and immunological markers. The most frequently studied variables were (i) the effects of probiotic administration on the composition of the microbiota and (ii) colonization with probiotics. Among microbial metabolites, mostly faecal SCFAs were analyzed. The authors analyzed also markers of gut-barrier integrity—mostly LPS and different cytokines as well as inflammatory markers. CRP measured in few studies can be considered as an inflammatory marker as well as a gut integrity marker. Based on the results obtained, no definite association can be found between the use of probiotics, microbiota changes, modulation of the immune system, and either presence or lack of clinical effects (Table 2 and Table S6). Of note, the results cannot be subjected to meta-analysis due to very diverse methods used to analyze the microbiota. Therefore, the results are difficult to compare. For this reason, in order to fully assess the causal relationship between the microbiota and the function of the immune system and gut-integrity markers with relation to cardiovascular risk prevention, a multifactorial analysis should be performed, which was not performed in the works described in this systematic review. In only one study, the correlation between microbiota changes and cardiovascular risk factors was demonstrated [48]; however, in this study, no preventive outcome of probiotics administration was observed. In addition, the results of metabolomic studies did not contribute to elucidation of the mechanism of action of probiotics studied. Therefore, it cannot be determined whether the effect of probiotics in cardiovascular risk prevention is related to their effect on microbiota or the immune system or gut-barrier function. The relationship observed in some studies is rather based on association and not causation. We conclude that mechanistic studies should be an important point in analysis of probiotics/synbiotics efficacy.

### Limitations

Several limitations of this meta-analysis need to be underlined. These include (i) a relatively small number of high-quality double-blinded studies comparing probiotic intervention to controls with a wide range within the number of participants preceded by no sample size calculations; (ii) heterogeneous study inclusion criteria (various age, profession of participants, and dietary and physical activity add-on interventions), and (iii) various type of strains and duration of probiotic intervention. In studies incorporated into the present meta-analysis, the association between the probiotic effect in relation to supplement dose and treatment duration was not analyzed. At last, most of the studies were financed by the industry and include products combined with different ingredients. These all are confounding factors for probiotic efficacy, which may have resulted in some publication bias as evaluated by Eagerr’s test and funnel plots [115]. Consequently, in order to draw some evidence-based conclusions and to give some guidelines regarding probiotic intake in healthy adults, strict inclusion criteria and homogenous intervention protocols are needed. Lastly, during meta-analysis, we did not use intent-to-treat data but adopted per-protocol evaluation as the majority of studies reported on that. We could have introduced potential bias during the review process and could have missed studies not clearly aimed at reducing cardiovascular risk but possibly reporting such outcomes.

## 5. Conclusions

Probiotics may counteract some CMRF (e.g., BMI and waist circumference) in clinically healthy participants. Overweight/obese persons might benefit from the reduction of total cholesterol serum concentration. Poor quality of probiotic-related trials make systematic reviews and meta-analyses difficult to conduct and draw exact conclusions. “Gold standard” methodology in probiotic studies awaits further development.

## Figures and Tables

**Table 1:** Study characteristics.

No.	Study Description				Intervention			Study Characteristisc				
	Reference/Year/Country/Sponsorship	Blinding/Crossover (Y/N)/Multiarm > 2	Focus on	ROB	Form/Probiotic Strain/Prebiotic	Probiotic Dose CFU	Duration of Probiotic Administration (Days)/Comparator	N Total Randomized and Allocated to Intervention/Analyzed	Age Years (Mean ± SD)	Males (n/%)	BMI Baseline (kg/m^2^): Probiotic Group (Mean ± SD)	BMI Baseline (kg/m^2^): Control group (Mean ± SD)
1	Agerbaek et al./1995/Denmark/Industry [29]	DB/N/N	Lipoprotein levels	2	Naturally fermented milk/*Enterococcus faecium* (1 strain), *Streptococcus termophilus* (2 strains)	Daily: *Enterococcus* faecium 4 × 10^10^ ; *Streptococcus termophilus* 1.4 × 10^11^	42/chemically fermented milk	58/57	44 (±ND)	57/100	24.3 (±2)	24.1 (±1.7)
2a	Agerholm-Larsen et al./2000/Denmark/Industry [30]	DB/N/Y: 5 arms: 3 probiotic groups and 2 placebo groups (3 probiotic and PBO tablet arms were analyzed)	Risk factors for cardiovascular disease	2	Yoghurt/2 strains of *Streptococcus thermophilus* and 2 strains of *Lactobacillus acidophilus*	3 days/week: *Streptococcus thermophilus* 4.5 × 10^10^, *Lactobacillus acidophilus* 9 × 10^9^	56/placebo tablets	2626	38.49 (±2.58)	7/26.92	30 (±2.8)	29.9 (±3.48)
2b					Yoghurt/2 strains of *Streptococcus thermophilus* and 1 strain of *Lactobacillus rhamnosus*	3 days/week: *Streptococcus thermophiles* 3.6 × 10^11^, *Lactobacillus rhamnosus* 9 × 10^10^	56/placebo tablets	2424	38.07 (±2.77)	7/29.17	30.2 (±2.62)	29.9 (±3.48)
2c					Yoghurt/1 strain of *Enterococcus faecium* and 2 strains of *Streptococcus termophilus*	3 days/week: *Enterococcus faecium* 2.7 × 10^10^, *Streptococcus thermophilus* 4.5 × 10^11^	56/placebo tablets	26/26	37.99 (±2.54)	7/26.92	30.1 (±2.4)	29.9 (±3.48)
3	Ahn et al./2015/South Korea/Non-industry [31]	DB/N/N	Triglyceride level and fasting plasma metabolome	2	Powder/*Lactobacillus curvatus* HY7601, *Lactobacillus plantarum* KY1032	Daily: *Lactobacillus curvatus* HY7601 5 × 10^9^ and *Lactobacillus plantarum* KY1032 5 × 10^9^	84/placebo powder	92/92	53.4 (±8.38)	30/32.61	24.7 (±2.91)	24.9 (±2.26)
4	Ahn et al./2015a/South Korea/Non-industry [32]	DB/N/N	Triglyceride and apolipoprotein A-V levels	2	Powder/*Lactobacillus curvatus* HY7601, *L. Plantarum* KY1032	Daily: *Lactobacillus curvatus* HY7601 0.5 × 10^10^ and *Lactobacillus plantarum* KY1032 0.5 × 10^10^	84/placebo powder	128/121	52.87 (±9.02)	33/27.27	24.9 (±3.2)	24.8 (±2.62)
5a	Andrade and Borges/2009/Portugal/industry [33]	DB/Y/N	Plasma lipids concentration	0	Fermented milk/*L. Acidophilus* 145 and *Bifidobacterium longum* BB536	Daily: *Lactobacillus acidophilus* 145 5.25–7.88 × 10^10^ and *Bifidobacterium longum* BB536 1.01–3.75 × 10^10^	28-7 washout-28/regular yoghurt)	41/34	35.44 (±11.17)	0/0	Baseline: Group probiotic-placebo 24.6 (±3.5) Group placebo-probiotic 24.9 (±3.40)	
5b												
6	Bjerg et al./2015/Denamark/industry [34]	DB/N/N	Blood lipids, fatty acids levels and stearoyl-coa desaturase−1 (SCD1) activity	3	Capsules/*L. Casei* W8	Daily: 1 × 10^10^	28/placebo capsules contained rice flour	70/64	Range: 20–45	34/48.57	23.7 (±)	23.7 (±)
7	Boesmans et al./2018/Belgium/Non-industry [35]	DB/Y/N	Blood parameters, fecal microbiota composition and metabolites	7	Capsules/*Butyricicoccus pullicaecorum* 25-3T	Daily: 1 × 10^8^	28-21 washout-28-21 washout/placebo capsules	30/28	Group probiotic-placebo 32 (26–45) Group placebo- probiotic 28 (25–33) 30(±ND) Albo Range: 25–45	14/46.67	Baseline: Group probiotic-placebo 23.6 (±2.1) Group placebo-probiotic 22.1 (±1.9)	
8	Brahe et al./2015/Multicenter/Academic/industry [36]	SB/N/Y 3 arms: 1 probiotic 1 prebiotic and 1 placebo group (Probiotic and placebo groups were analyzed)	The gut microbiota composition, fecal SCFA concentration and metabolic risk markers in obesity	3	Powder/*Lactobacillus paracasei* F19	Daily: 9.4 × 10^10^	42/placebo powder	39/35	59.92 (±6.09)	0/0	34.2 (±3.1)	34.3 (±3.8)
9	Bukowska et al./1998/poland/Non-industry [37]	DB/N/N	Metabolic parmeteres	2	Drink/*Lactobacillus plantarum* 299 v/oat fibers	Daily: *Lactobacillus plantarum* 299v 1 × 10^10^ and 160 mg oat fibers	42/control drink (rose hip drink)	30/30	42.65 (±2.57)	30/100	26.6 (±3.7)	25.9 (±2.6)
10	Chang et al./2011/South Korea/Non-industry/industry [38]	DB/N/N	Metabolic parameters	2	The functional yogurt: starters: *S. thermophilus*, *L. acidophilus*, *B. infantis*; probiotics: *Enterococcus faecalis* FK-23, *Bifidobacterium breve*; fibersol-2 (resistant maltodextrin); pine needle extract; whey protein hydroxylate; Rice germ extract powder; Yucca schidigera and Quillaja saponaria extract	ND	87/(control yoghurt with starters)	103/101	36.78 (±9.45)	31/30.69	22.63 (±3.26)	22.13 (±2.8)
11a	Cox et al./2014/Multicenter/industry [39]	DB/N/Y: 3 arms: 2 probiotic groups and 1 placebo group	Routine haematology and clinical chemistry measures	2	Powder/*Bifidobacterium animalis subsp. Lactis* Bl-04,	Daily: 2 × 10^9^	150/placebo powder	87/84	40.43 (±13.72)	44/52.38	24.6 (±3.2)	24.1 (±3.1)
11b					Powder/*Lactobacillus acidophilus* NCFM, *Bifidobacterium animalis* subsp. *Lactis* Bi-07	Daily: total dose1 × 10^10^ (equal amount of each strain)		91/90	38.14 (±11.17)	42/50	24.4 (±3.8)	24.1 (±3.1)
12	De Roos et al./2017/netherlands/Non-industry [40]	DB/N/N	Migraine symptom reduction, an effect on intestinal permeability and inflammation markers	4	Powder/*Bifidobacterium bifidum* W23, *Bifidobacterium lactis* W52, *Lactobacillus acidophilus* W37, *Lactobacillus brevis* W63, *Lactobacillus casei* W56, *Lactobacillus salivarius* W24, *Lactococcuslactis* W19, *Lactococcus lactis* W58	Daily: 5 × 10^9^	84/placebo powder	63/60	40.07, Range: 18–70	4/6.67	24.2 (±NA)	25.6 (±NA)
13	Fabian et al./2006/Austria/industry [41]	ND/N/N	Plasma lipid profile	0	Yoghurt/starter cultures: *Streptococcus thermophilus* and *Lactobacillus bulgaricus*; probiotic: *L. casei* DN-114 001	Daily: Days 1–14: 3.6 × 10^10^/Days 15–28: 7.2 × 10^10^	28/placebo regular yoghurt	34/33	24 ± 2.56	0/0	20.7 (±3)	21 (±2.7)
14	Gleeson et al./2012/United Kingdom/Industry [42]	DB/N/N	Incidence of upper respiratory tract infections (URTI) and mucosal immune markers	1	Powder/*Lactobacillus salivarius*	Daily: 2 × 10^10^	112/placebo powder	66/54	23.9 (±4.7) On PRO age 25 ± 5 years PBO age 24 ± 4 years	28/42.42 (during randomization)	24.2 (±3.4)	23.2 (±2.7)
15	Gohel et al./2016/india/Non-industry [43]	DB/Y/N	Calcium level and haematological parameters	3	Fermented milk/*Lactobacillus helveticus* MTCC 5463	Daily: min. 2 × 10^10^	28-28 washout-28-28 washout/placebo milk	76/59	68.93 (±4.1)	38/50	ND	ND
16	Gomes et al./2017/Brazil/Non-industry [44]	DB/N/N	Body composition, lipid profile, endotoxemia, inflammation, and antioxidant profile	5	Powder/*Lactobacillusacidophilus* LA-14, *Lactobacillus casei* LC-11, *Lactococcus lactis* LL-23, *Bifidobacterium bifidum* BB-06, and *Bifidobacteriumlactis* BL-4	Daily: 2 × 10^10^ (equal amount of each strain)	56/(placebo powder)	60/43	Range: 20–59	0/0	31.7 (±3.9)	33.34 (±4.69)
17	Greany et al./2008/USA/Non-industry/industry [45]	SB/N/N	Plasma lipids	2	Capsules/*Lactobacillus acidophilus* DDS-1, *Bifidobacterium longum* UABL-14/FOS	Daily: *L. acidophilus* DDS-1: 3.75 × 10^9^; *B. longum* UABL-14: 3.75 10^9^ plus 30–45 mg FOS	52/placebo capsules	64/55	26.77 (±5.07)	22/40	24.1 (±3.1)	22.8 (±3.5)
18	Guillemard et al.2010/Multicenter/Industry [46]	DB/N/N	Incidence of respiratory and gastrointestinal common infectious diseases (cids) and immune functions	3	Fermented dairy drink/*Lactobacillus casei* DN-114 001	Daily: 2 × 10^10^	84/placebo diary drink)	1000/962	32.15 (±8.91)	435/43.5 (during randomization)	24 (±2.8)	24.2 (±2.9)
19	Hatakka et al./2008/Finland/Industry [47]	DB/Y/N	Serum cholesterol and triglyceride levels	2	Capsules/*L. rhamnosus* LC705 and P. *freudenreichii* JS	Daily: *L. rhamnosus* LC70 5 2 × 10^10^; *P. freudenreichii* JS 2 × 10^10^	28-28/(placebo capsules)	38/38	42 (±7.28)	38/100	Baseline: Group probiotic-placebo 25.2 (±3.4) Group placebo-probiotic 24.5 (±2.6)	
20a	Hibberd et al./2019/Multicenter/industry [48]	DB/N/Y: 4 arms: 1 probiotic group, 1 synbiotic group and 2 control groups (Prebiotic and synbiotic and placebo arms were analyzed)	Body fat mass and obesity-related markers	1	Powder/*Bifidobacterium animalis* subsp. *Lactis* 420	Daily: *Bifidobacterium animalis* subsp. *Lactis* 420, 1 × 10^10^	168/placebo powder	61/61	48.63 (±10.09)	17/27.87	30.9 (±1.9)	31 (±2.2)
20b					Powder/*Bifidobacterium animalis* subsp. *Lactis* 420 and Litesse Ultra (refined polydextrose)	Daily: *Bifidobacterium animalis* subsp. *Lactis* 420, 1 × 10^10^ + 12 g Litesse Ultra (refined polydextrose)		73/73	47.69 (±9.85)	16/21.92	31.2 (±2)	31 (±2.2)
21a	Higashikawa et al./2016/Japan/Non-industry [49]	DB/N/Y: 3 arms: probiotic group, killed bacteria group and control group	Body fat and body weight	6	Powder/*Pediococcus pentosaceus* LP28 (live)	Daily: LP28 1 × 10^11^	84/placebo powder	41/41	52.65 (±11.7)	15/36.58	26.84 (±1.15)	27.37 (±1.43)
21b					Powder/*Pediococus pentosaceus* LP28 (heat-killed)			41/41	54.18 (±10.89)		27.1 (±1.24)	
22a	Ibrahim et al./2018/Malaysia/Non-industry/industry [50]	ND/N/Y: 4 arms: 2 sedentary groups: probiotic group, placebo group; 2 circuit training groups: probiotic, placebo	Muscular strength and power and cytokine responses	1	Powder/*Lacidophilusacidophilus* BCMC 12130, *L. casei* BCMC 12313, *L. lactis* BCMC 12451, *Bifidobacterium bifidum* BCMC 02290, *B. infantis* BCMC 02129, and *B. longum* BCMC 02120)	Sedentary groups Daily: 6 × 10^10^	84/placebo powder	24/20	22.5 (±1.66)	24/100	21.8 (±3.4)	21.1 (±2.8)
22b						Circuit training groups Daily: 6 × 10^10^	84/placebo powder plus circuit training	24/21	21.43 (±2.53)		22.1 ±3.4	21.1 ±2.7
23	Inoue et al./2018/Japan/Non-industry [51]	DB/N/N	Cognitive function, mental state, body composition, and bowel movement were measured	4	Powder/*B. longum* BB536, *B. infantis* M-63, *B. breve* M-16V and *B. breve* B-3	Daily: 1.25 × 10^10^ of each strain	84/placebo powder+	39/38	70.3 (±3.1)	14/36.82	24 (±2.8)	23 (±2.7)
24	Ito et al./2017/Japan/Non-industry [52]	DB/N/N	Serum lipids level	5	Fermented milk/*Streptococcus thermophilus* YIT 2001	Daily: ≥1 × 10^11^	84/placebo non-fermented milk	60/59	47.35 (±8.25)	30/50.84	22.4 (±2.8)	23.3 (±2.8)
25	Ivey et al./2014/Australia/Non-industry/Industry [53]	DB/N/Y: 4 arms: 2 probiotic yoghurt groups: probiotic capsules group, placebo group; 2 control milk: probiotic capsules, placebo (probiotic yoghurt, control milk	Biomarkers of glycaemic control	4	Yoghurt and capsules/*Lactobacillus acidophilus* La5, *Bifidobacterium animalis* subsp *lactis* Bb12	Daily: 3 × 10^9^ (both yoghurt and capsules)	42/probiotic yoghurt placebo capsules,	77/77	68.4 (±8.25)	50/64.93	30.6 (±3.8)	30.2 (±4.3)
					Capsules/*Lactobacillus acidophilus* La5, *Bifidobacterium animalis* subsp *lactis* Bb12		42/control milk, placebo capsules	79/79	65.05 (±7.79)	46/58.23	30.8 (±3.5)	30.8 (±3.5)
26	Ivey et al./2015/Australia/Non-industry/industry [54]	DB/N/Y: 4 arms: 2 probiotic yoghurt groups: probiotic capsules group, placebo group; 2 control milk groups: probiotic capsules, placebo	Blood pressure and serum lipid profile	4	Yoghurt and capsules/*Lactobacillus acidophilus* La5, *Bifidobacterium animalis* subsp *lactis* Bb12	Daily: 3 × 10^9^ (both yoghurt and capsules)	42/placebo capsules, control milk	Probiotic yoghurt 77/77	68 (±8.34)	50/64.93	31 (±4)	30 (±4)
								Control milk 79/79	65 (±7.52)	46/58.23	31 (±4)	31 (±4)
27	Jones et al./2016/Canada/industry [55]	DB/N/N	Blood cholesterol concentration	5	Capsules/*Lactobacillus. reuteri* NCIMB 30242	Daily: min. 4.0 × 10^9^	63/(placebo capsules)	131/127	49.09 (±13.57)	55/43.31	26.83 (±3.05)	27.62 (±2.81)
28	Kadooka et al./2010/Japan/ND [56]	DB/N	Abdominal adiposity, body weight and other body measures in adults with obese tendencies	2	Fermented milk/*Lactobacillus gasseri* SBT2055 (LG2055)	Daily: 1 × 10^11^	84/placebo fermented milk	87/87	48.76 (±9.21)	59/67.82	27.5 (±1.67)	27.2 (±1.69)
29a	Kadooka et al./2013/Japan/Non-industry [57]	DB/N/Y: 3 arms: lower dose probiotic group, higher dose probiotic group, control group	Abdominal adiposity, anthropometric measures and body composition	2	Fermented milk/*Lactobacillus gasseri* SBT2055	Daily: 2 × 10^9^	84/fermented placebo milk	139/139	47.15 (±7.21)	69/49.64	27.5 (±1.9)	27.2 (±1.9)
29b						Daily: 2 × 10^8^		141/141	47.29 (±7.21)	72/51.06	27.2 (±1.8)	27.2 (±1.9)
30	Kawase et al./2000/Japan/Non-industry [58]	SB/N/N	Serum lipid level	2	Fermented milk/*Lactobacillus casei* subsp. *casei* TMC0409, *Streptococcus thermophilus* TMC1543	Daily: L. casei TMC0409 2.44 × 10^11^, S. thermophilus TMC1543 1.04 × 10^10^	59/fermented placebo milk	20/20	40.1 (±ND)	20/100	ND	ND
31a	Kim et al./2018/South Korea/Non-industry/Non-industry [59]	DB/N/Y: 3 arms: lower dose probiotic group, higher dose probiotic group, control group	Adiposity	5	Capsules/*Lactobacillus gasseri* BNR17	Daily: 1 × 10^9^	84/placebo capsules	60/60	38.7 (±11.76)	20/33.3	27.9 (±1.07)	28.6 (±1.96)
31b						Daily: 1 × 10^10^		60/60	38 (±10.4)	21/35	28.8 (±2.24)	28.6 (±1.96)
32	Kim et al./2017/South Korea/Non-industry [60]	DB/N/N	Adiposity parameters and metabolomic profile	5	Powder/*L. curvatus* HY7601 and *L. Plantarum* KY1032	Daily: *Lactobacillus curvatus* HY7601 2.5 × 10^9^, *Lactobacillus plantarum* KY1032 2.5 × 10^9^	84/placebo powder	120/66	38.99 (±1.93)	ND	26.6 (±1.3)	27.1 (±1.57)
33	Klein et al./2008/Germany/Non-industry [61]	DB/Y/N	Blood lipids, faecal microbiota, and immunological parameters	3	Yoghurt/*B. lactis* DGCC420, *L. acidophilus* 74-2	Daily:*B. lactis* 9 × 10^8^; *L. acidophilus* 74-2 2.79 × 10^11^	35-35/placebo yoghurt	26/26	25 (±3)	13/50	Baseline: Group probiotic-placebo 21.3(±2.1) Group placebo-probiotic21.5(±2.0)	
34	Lambert et al./2017/Denmark/industry/Non-industry [62]	DB/N/N	Anthropometric data, lipids level, and menopausal symptoms	4	Drink/Heterogeneous culture of probiotic lactic acid bacteria in red clover drink	ND	84/placebo water based drink	62/59	52.34 (±3.66)	0/0	26.02 (±5.38)	25.45 (±3.34)
35a	Lee et al./2017/USA/Non-industry [63]	Partially SB/Y/Y: 4 arms: (1) placebo yogurt, (2) yogurt with probiotic added pre-fermentation, (3) yogurt with probiotic added post-fermentation, and (4) probiotic capsules	Blood lipids level and fecal excretion of scfas	3	Yoghurt, capsules/*Bifidobacterium animalis* subsp. *Lactis* BB-12^®^	Daily: 3.16 × 10^9^	Each treatment period 28-washout 14/placebo capsules	36/30	28.2 (±6.4)	11/36.67	All crossover groups 24.2 (±2.6)	
35b												
35c												
36	Lin et al./1989/USA/industry [64]	DB/Y/N	Serum lipids level	4	Tablets/*L. acidophilus* (ATCC 4962) and *L. Bulgaricus* (ATCC 33409)	Daily: 8 × 10^6^	42-21 washout-42/placebo tablets	334/334	ND	ND	ND	
37	Macfarlane et al./2013/United Kingdom/Non-industry [65]	DB/Y/N	Colonic microbiota composition, immune function and health status	5	Capsules/*Bifidobacterium longum*/Powder/Synergy I mixture of inulin and oligofructose (DP2-60)	Daily: 4 × 10^11^ *B. longum* + 12 gof prebiotic)	28-28 washout-28/placebo capsules and powder	47/43	71.9 (±5.4)	21/48.83	All crossover groups 26.9 (±4.2)	
38	Madjd et al./2016/Multicenter/Non-industry [66]	SB/N/N	Body weight and cardiometabolic risk factors	3	Yoghurt/*Lactobacillus acidophilus* LA5, *Bifidobacterium lactis* BB12	ND	84/low fat yoghurt	89/89	31.98 (±6.88)	0/0	32.14 (±3.2)	32.05 (±3.94)
39a	Mohammad Moradi et al./2015.Iran/non-industry [67]	TB/N/Y: 3 arms: (1) probiotic cheese and extract of chicory root, (2) probiotic cheese, and (3) control	Lipid profile	5	Cheese/*Lactobacillus acidophilus* LA5, *Bifidobacterium lactis* BB12and raw chicory root	ND	49/no intervention	120/120	37.55 (±15.97)	60/50	22.38 (±2.01)	22.14 (±0.97)
39b					Cheese/*Lactobacillus acidophilus* LA5, *Bifidobacterium lactis* BB12			120/120	39.4 (±15.92)	60/50	21.94 (±2.19)	22.14 (±0.97)
40	Naruszewicz et al./2002/poland/Non-industry [68]	DB/N/N	Lipid profiles, inflammatory markers, and monocyte function	3	Drink/*Lactobacillus plantarum* 299v	Daily: 2 × 10^10^	42/placebo drink	36/36	42.3 (±3.9)	18/50	24.8 (±4.8)	25.8 (±3.7)
41	Nishiyama et al./2018/Japan/industry [69]	DB/N/N	Immunity and metabolic syndrome parameters	2	Yoghurt/*L. lactis* 11/19-B1, *B. Lactis* BB-12	No data available	56/placebo yoghurt	79/76	42.35 (±11.15)	29/38.15	ND	ND
42	Nova et al./2011/Spain/Non-industry [70]	DB/N/N	Self-perceived gastrointestinal well-being and immunoinflammatory status	3	Tablets/*L. Acidophilus La5, B. animalis Ssp. Lactis Bb-12, Lactobacillus delbrueckii ssp. bulgaricus, Streptococcus thermophilus*, and *Lactobacillus paracasei* ssp. *paracasei* and FOS	Daily: 2.4 × 10^9^	42/placebo tablets with no probiotics)	37/36	Range 25–45	16/44.4	23.74 (±2.19)	23.06(±2.32)
43	Ostan et al./2015/Multicenter/Non-industry [71]	ND/N/Y: from 4 arms only probiotic and control arm were analysed	Inflammageing, oxidative stress, and gut microbiota composition	3	Capsules/*Lactobacillus paracasei, L. plantarum, L. acidophilus, L. delbrueckii* subsp *bulgaricus, bifidobacterium longum, B. breve, B. infantis, Streptococcus thermophilus*	Daily: 2.24 × 10^11^	56/Ristomed diet alone	69/59	70.4 (±3.9)	58/46.4	26.7(±3.8)	26.9(±3.4)
44	Osterberg et al./2015/USA/Non-industry [72]	DB/N/N	Body and fat mass, insulin sensitivity, and skeletal muscle substrate oxidation	3	Powder/*Streptococcus thermophilus* DSM24731, *Lactobacillus acidophilus* DSM24735, *Lactobacillus delbrueckii* ssp. *Bulgaricus* DSM24734, *Lactobacillus paracasei DSM24733, Lactobacillus plantarum DSM24730, Bifidobacterium longum DSM24736, Bifidobacterium infantis DSM24737*, and *Bifidobacterium breve* DSM24732	Daily: 9 × 10^11^	28/placebo powder	20/20	22.6 (±3.59)	20/100	23.9 (±2.7)	23.2 (±1.99)
45a	Rajkumar et al./2014/india/Non-industry [73]	SB/N/Y: 4 arms: placebo, probiotic, omega-3 fatty acid, omega-3 fatty acid + probiotic (probiotics and placebo arms were analyzed)	Insulin sensitivity, blood lipids, and inflammation	6	Capsules/*Bifidobacterium longum, B. infantis, B. breve, Lactobacillus acidophilus, L. paracasei, L. delbrueckii* subsp. *bulgaricus, L. Plantarum, Streptococcus salivarius* subsp. *thermophilus*;	Daily: 1.13 × 10^11^	42/placebo capsules	30/30	49 (40-60)	30/50	28.79 (Range: 27–30)	
45b					Capsules/*Bifidobacterium longum, B. infantis, B. breve, Lactobacillus acidophilus, L. paracasei, L. delbrueckii* subsp. *bulgaricus, L. plantarum, Streptococcus salivarius* subsp. *thermophilus, Omega 3 fatty acids*	Daily: 1.13 × 10^11^ + Omega 3 (360 mg EPA and 240 mg DHA)						
46	Sadrzadeh-Yeganeh et al./2010/Multicenter/industry [74]	TB/N/Y: 3 arms: placebo yoghurt, probiotic yoghurt, and no intervention (probiotic and placebo arms were analysed)	Lipid profile	2	Yoghurt/Placebo: *Lactobacillus bulgaricus and Streptococcus thermophilus*. Probiotic: Placebo + *Lactobacillus acidophilus* La5, *Bifidobacterium lactis* Bb12	ND	42/placebo yoghurt,	60/59	34.06 (±5.74)	ND	Placebo yoghurt 23 (±2.4) Probiotic yoghurt 24 (±2.4)	
47	Sanchez et al./2014/Multicenter/industry [75]	DB/N/N	Weight loss and weight maintenance	4	Capsules/*Lactobacillus rhamnosus* CGMCC1.3724 and mix of oligofructose and inulin	2 Daily: 3.24 × 10^8^ and 600 mg of a mix of oligofructose and inulin (70:30, v/v)	168/placebo capsules	125/93	36 (±79.06)	48/38.4	33.8 (±25.98)	33.3 (±25.39)
48a	Savard et al./2011/Canada/industry [76]	DB/N/Y: 3 arms: lower dose of probiotics and green tea extract, higher dose of probiotic and green tea extract, and placebo	Fecal bacterial counts of *Lactobacillus acidophilus* LA-5 and *Bifidobacterium animalis subsp. Lactis* BB-12 and lipid profile	3	Yoghurt/Starters: *Lactobacillus delbrueckii subsp. Bulgaricus* and *Streptococcus thermophilus*; Probiotics: *Bifidobacterium animalis* subsp. *Lactis* BB-12, *Lactobacillus acidophilus* LA-5 and green tea extract	1 arm, daily: *Bifidobacterium animalis* subsp. *Lactis* BB-12 1 × 10^9^, *Lactobacillus acidophilus* LA-5 1 × 10^9^ and 40 mg of green tea extract	28/placebo yoghurt containing no starter culture, no probiotic, and no green tea extract	40/38	32 (±11.9)	12/30	22.8 (±3.8)	23.8 (±4.1)
48b						2 arm, daily: *Bifidobacterium animalis* subsp. *Lactis* BB-12 1 × 10^10^, *Lactobacillus acidophilus* LA-5 1 × 10^9^ and 40 mg of green tea extract		38/36	33.27 (±12.37)	12/31.6	23.7 (±2.7)	23.8 (±4.1)
49	Simon et al./2015/Multicenter/Non-industry [77]	DB/N/Y: 4 arms: lean group: 1) probiotics, 2) placebo; obese group: 1) probiotics, 2) placebo	Insulin sensitivity	4	Capsules/*Lactobacillus reuteri* SD5865	Daily: 2 × 10^10^	28/placebo capsules	Lean: 11/11 Obese:10/10	50 (±7)	Lean: 5/45 Obese:5/50	Lean: 23.6 (±6 1.7) Obese: 35.5±4.9	
50	Simons et al./2006/Australia/Industry [78]	DB/N/N	LDL cholesterol and other lipid fractions level	3	Capsules/*Lactobacillus fermentum*	Daily: 8 × 10^9^	70/placebo capsules	46/44	51.5 (±11.5)	16/36.36	27 (±5.7)	24.4 (±3.7)
51a	Stenman et al./2016/Multicenter/industry [79]	DB/N/Y: 4 arms: (1) placebo, (2) prebiotic, (3) probiotic (4) synbiotic (probiotic, synbiotic vs. Placebo arms were analyzed)	Body fat mass and other obesity-related parameters	4	Powder/Probiotic: *Bifidobacterium animalis ssp. Lactis 420* (B420); Prebiotic: polydextrose; Synbiotic: combination of above	Daily: B420 1 × 10^10^;	186/placebo powder,	112/61	48.67 (±10.23)	17/27.86	30.9 (±1.9) 31.2 (±2)	31 (±2.2) 31 (±2.2)
51b						Synbiotic: B420 1 × 10^10^ +; polydextrose 12g		113/73	47.75 (±9.75)	16/21.92		
52a	Szulińska et al./2018/Poland/Non-industry [80]	DB DB/N/Y: 3 arms: (1) lower dose probiotic, (2) higher dose probiotic, and (3) placebo	Functional (primary endpoint) and biochemical parameters (secondary endpoint) of endothelial dysfunction	5	Powder/*Bifidobacterium bifidum* W23, *Bifidobacterium lactis* W51, *B. Lactis* W52, *Lactobacillus acidophilus* W37, *Lactobacillus brevis* W63, *Lactobacillus casei* W56, *Lactobacillus salivarius* W24, *Lactococcus lactis* W19, *Lactococcus lactis* W58	Daily: 1 × 10^9^	84/placebo powder	54/48	57.55 (±7)	0/0	36 (±5.2)	36.1 (±4.37)
52b						Daily: 2.5 × 10^10^		54/47	56.94 (±7.28)		36.57 (±5.95	36.1 (±4.37)
53a	Szulińska et al./2018a/Poland/Non-industry [81]	DB/N/Y: 3 arms: (1) lower dose probiotic, (2) higher dose probiotic, (3) placebo	Cardiometabolic biochemical parameters, and lipopolysaccharide levels	5	Powder/*Bifidobacterium bifidum W23, Bifidobacterium lactis W51, Bifidobacterium lactis W52, Lactobacillus acidophilus W37, Lactobacillus brevis W63, Lactobacillus casei W56, Lactobacillus salivarius W24, Lactococcus lactis W19*, and *Lactococcus lactis W58*.	Daily: 1 × 10^9^	84/placebo powder	54/48	57.55 (±7)	0/0	36 (±5.2)	36.1 (±4.37)
53b						Daily: 2.5 × 10^10^		54/47	56.94 (±7.28)		36.57 (±5.95)	36.1 (±4.37)
54a	Tenore et al./2019/Italy/Non-industry [82]	DB/N/Y: 3 arms: lactofermented Annurca apple puree, probiotic, unfermented apple puree	Lipid profile and oxidative metabolites level	6	Capsules/*Lactobacillus rhamnosus* LRH11, *Lactobacillus plantarum* SGL07	1 arm, daily: 3 × 10^8^	112/Annurca apple puree with no probiotics	54/42	46.65 (±10.36)	30/71.43	≤30	
54b					Annurca apple puree fermeted with *Lactobacillus rhamnosus* LRH11, *Lactobacillus plantarum* SGL07	Daily: probiotics 3.0 × 10^8^ + Annurca apple puree 125 2 arm, Daily: probiotics 3.0 × 10^8^ + Annurca apple puree 125 g		53/41	45.64 (±10.51)	31/75.61		
55	Trautvetter et al./2012/Germany/industry [83]	DB/Y/N	Intestinal colonisation of *L. Paracasei* and blood cholesterol level	3	Yoghurt/*Lactobacillus paracasei* LPC37 and bread containing pentacalcium hydroxy-triphosphate	Daily: probiotic 1 × 10^12^ and calcium 1 g	28-28 washout-28/placebo yoghurt and bread)	32/32	25 (±5)	ND	22 (±3)	
56a	Usinger et al./2010/Denmark/Industry [84]	DB/N/Y:3 arms: 150mL probiotic milk, 300 mL of probiotic milk, chemically acidifies milk	Blood pressure	5	Milk/*Lactobacillus helveticus* Cardi-04	Daily 150 mL of milk fermented with probiotic strain (dose not available) and contains 1.25 mg Val–Pro–Pro (VPP) and 0.55 mg Ile–Pro–Pro (IPP)	56/chemically acidified milk	47/45	53.3 (±7.41)	36/60	26 (±4)	26 (±4)
56b						Daily 300 mL of milk fermentem with probiotic strain (dose not available) and contains 2.5 mg Val–Pro–Pro (VPP) and 1.1 mg Ile–Pro–Pro (IPP)		47/44		28/46.67	27 (±4)	26 (±4)
57	Valentini et al./2015/Multicenter/Non-industry [85]	SB/N/N	Biomarkers of inflammation, nutrition, oxidative stress and intestinal microbiota	4	Capsules/*Bifidobacterium infantis* DSM24737, *Bifidobacterium longum* DSM24736, *Bifidobacterium breve* DSM24732, *Lactobacillus acidophilus* DSM24735, *Lactobacillus delbruckii ssp.bulgaricus* DSM 27734, *Lacctobacillus paracasei* DSM 24733, *Lactobacillus plantarum* DSM24730, *Streptococcus thermophilus* DSM 24731	Daily: 2.24 × 10^11^	56/Ristomed diet	69/62	70.1(±3.9)	29/46.77	26.8 (±3.59)	
58	Välimäki et al./2012/finland/Non-industry [86]	DB/N/N	Oxidized LDL lipids, serum antioxidant potential (s-TRAP) and serum antioxidants (s-α-tocopherol, s-γ-tocopherol, s-retinol, s-β-carotene, and s-ubiquinone-10)	4	Milk drink or capsules/*Lactobacillus rhamnosus* GG	Daily: drink 4x × 10^10^ or capsules 1 × 10^10^	84/placebo drink or capsules	141/119	40 (23–69)	105/88.2	22 (Range: 18–26)	
59	Venkataraman et al./2018/India/ND [87]	SB/N/N	Blood glycemic markers concentration	2	Capsules/*Lactobacillus salivarius* UBLS22, *Lactobacillus casei* UBLC 42, *Lactobacillus plantarum* UBLP 40, *Lactobacillus acidophilus* UBLA 34, *Bifidobacteriu breve* UBBR 01, *Bacillus coagulans Unique*-IS2/FOS	3.0 × 10^8^ cfu/30 × 10^9^ CFU/capsule	84/placebo capsule	80/80	ND	ND	ND	ND
60	Xiao et al./2003/Japan/industry/Non-industry [88]	SB/N/N	Blood lipids level	2	Yoghurt/*Bifidobacterium longum* BL1	Daily: 3 × 10^10^	28/placebo yoghurt	32/32	43.85 (±8.05)	32/100	ND	ND
61a	Zarrati et al./2014/Iran/Non-industry [89]	DB/N/Y: 3 arms: probiotic yoghurt with low calorie diet (LCD), probiotic yoghurt without LCD, regular yoghurt with LCD	Body fat percentage, blood proinflammatory markers and cytokines content	3	Yoghurt/*Lactobacillus acidophilus* LA5, *Lactobacillus casei* DN001, *Bifidobacterium lactis* BB12 without LCD	Daily: 2 × 10^10^	56/regular yoghurt with LCD	50/50	35.5 (±9.27)	24/32	32 (±3.62)	33.9 (±6.73)
61b					Yoghurt/*Lactobacillus acidophilus* LA5, *Lactobacillus casei* DN001, *Bifidobacterium lactis* BB12 with lcd			50/50	36 (±9.07)		33.8 (±6.35)	33.9 (±6.73)

DB—double blind, SB—single blind, TB—triple blind, N—no, Y—yes, NA—not applicable, CFU—colony-forming units, ROB—risk of bias, SD—standard deviation, URTI—upper respiratory tract infection, CIDs—common infectious diseases, LDL—low-density lipoprotein, TRAP—total reactive antioxidant potential, EPA—eicosapentaenoic acid, DHA—decosahexaenoic acid, ND—not determined, PBO—placebo, PRO—probiotic, FOS—fructooligosaccharides.

**Table 2:** Microbiota and gut-barrier-related outcomes.

No.	Reference/Year/Country/Sponsorship	Microbiota	Microbiota Related (Metabolites)	Gut Barrier and Inflammatory Markers	Methods
1	Boesmans et al./2018/Belgium/Non-industry [35]	No impact on the microbiota richness and single genera abundances, only transcient gut colonization by probiotic strain used in the study	No influence on microbiota metabolic activity as well as on saccharolytic and proteolytic fermentation processes markers (SCFAs and dimethyl sulfide, p-cresol, indole, and the branched-chain fatty acids)	No influence on faecal calprotectin concentrations	NGS, GC-MS
2	Brahe et al./2015/Multicenter/Academic/Industry [36]	Probiotc group: alterations in faecal abundance of 2493 bacterial genes ≥ ↑ *Eubacterium rectale* and ↑*Ruminococcus torques*. Placebo group: altered faecal abundance of 7436 genes ≥ ↑*Roseburia hominis*, ↑two *Clostridiales*, ↑ one unknown species, ↓ *Eubacterium ventriosum*, and ↓ one unknown species.	No impact on faecal total SCFAs and butyric acid	No impact on lipopolysaccharide-(LPS)-binding protein and inflammatory markers (plasma high-sensitivity C-reactive protein (CRP), serum tumor necrosis factor-α (TNFα), and plasma interleukin (IL)-6	metagenomics, ethyl chloroformate NEFA method and GC
3	de Roos et al./2017/Netherlands/Non-industry [40]			No impact on zonulin concentration and intestinal permeability measured by means of lactulose-mannitol test; no changes of IL -6, IL-10, TNFα, and CRP	ELISA, GC
4	Hibberd et al./2019/Multicenter/Industry [48]	Probiotic group ↑*Akkermansia muciniphila*, ↑*Lactobacillus*, ↑ *Bifidobacterium* OTU, ↑*Akkermansia*, ↑ *Streptococcus*, ↑ S24-7, ↑*Methanobrevibacter*, ↑*Clostridiaceae spp.*, ↑Clostridium, ↑Phascolarctobacterium, ↑*Dialister*; ↓*Bacteroides*, ↓ *Erysipelotrichaceae spp.*, ↓ *Enterobacteriaceae spp.*, ↓ RF39 spp. *Bifidobacterium* was positively correlated to lean body mass (total, arms, legs, trunk, and android). *Paraprevotella* negatively correlated with fat mass. Synbiotic group The most pronounced changes of microbiota alterations in clustering analysis (long-term effect). Phylum Bacteroidetes: ↑ taxa: S24-7, *Barnesiellaceae spp.*, Parabacteroides, and Rickenellaceae spp.; ↓ taxa: Paraprevotella. Phylum Firmicutes: ↑ taxa: *Christensenellaceae*, *Ruminococcaceae spp.*, *Oscillospira*, *Phascolarctobacterium*, *Erysipelotrichaceae spp.* ↓ taxa: *Lactobacillus*, *Lactococcus*, *Turicibacter* *Streptococcus*, *Clostridiales spp.*, *Lachnospira* Phylum Actinobacteria: ↓ taxa: *Adlercreutzia*, *Collinsella*, *Eggerthella* Others: ↑*Methanobrevibacter*, ↑ *Akkermansia*; ↓ *Enterobacteriaceae spp.*, and ↓ RF39 spp. Christensenellaceae spp. abundance was correlated negatively to WHR and energy intake at baseline, and waist-area body fat and cholesterol markers were correlated to android fat, trunk fat, and lipid parameters *Christensenellaceae spp.* was positively correlated to the faecal branched-chain fatty acids (BCFAs), isobutyric acid, isovaleric acid, 2-methyl-butyric acid, and 3-methyl-2-oxovalerate, and to the plasma bile acids.	Probiotic group ↓ propionate Synbiotic group Metabolites: ↓ primary conjugated plasma bile acid glycocholic acid (GCA) ↓ secondary conjugated bile acids glycoursodeoxycholic acid (GUDCA) and taurohyodeoxycholic acid and tauroursodeoxycholic acid (THDCA + TUDCA). ↑ carbohydrates/polysaccharides PICRUSt: “Cellular Processes” and “Metabolism”-KEGG pathways differentially abundant from placebo group No significant changes in short-chained fatty acids (SCFA) or amino acids for any treatment group		NGS, NMR
5	Jones et al./2016/Canada/Industry [55]			Highly sensitive (hs) CRP was unchanged.	LC-MS, GC-MS
6	Klein et al./2008/Germany/Non-industry [61]	*L. acidophilus* and *B. lactis* elevation	No impact on SCFAs	↑ phagocytic activity as a marker for the unspecific cellular immune response	EUB-positive, DAPI-staining, FISH-based quantification, GC
7	Lee et al./2017/USA/Non-industry [63]		↑fecal acetate in control yoghurt group and in probiotic added before fermentation group; other SCFAs were unchanged.	CRP level was unchanged.	GC-MS
8	Macfarlane et al./2013/United Kingdom/Non-industry [65]	↑Actinobacteria, ↑ some species of Firmicutes,↑ total bifidobacterial population, ↑ *B. angulatum*, ↑ *B. longum*, ↑ *B. adolescentis*, ↑ *B. bifidum.* ↓Proteobacteria.↑ *Firmicutes/Bacteroidetes* ratio.	↑ butyrate, succinate, total acetate, propionate.	↓TNF-α	FISH, GC
9	Osterberg et al./2015/USA/Non-industry [72]	↑*Streptococcus thermophiles*, ↑ *Lactobacillus acidophilus*		No changes of LPS Binding Protein (LBP), LBP/sCD14, IL6, TNFα, hsCRP	qPCR
10	Rajkumar et al./2014/India/Non-industry [73]	Probiotic group and probiotic + omega-3 group ↑ total aerobes, ↑total anaerobes, ↑*lactobacillus*, ↑ *bifidobacteria*, ↑ streptococcus in the. Probiotic + omega-3 group significant effect on↑ *Bacteroides*, ↓coliforms, and ↓*E. coli.*		↓hsCRP	culture-dependent
11	Sanchez et al./2014/Multicenter/Industry [75]	Males: No changes in gut microbiota Females: ↓, *Lachnospiraceae* family↓ *Subdoligranulum* genus.	No change of β-hydroxybutyrate level.	No change of CRP and LPS level	NGS, ELISA
12	Savard et al./2011/Canada/Industry [76]	↑*B. animalis* subsp. *Lactis*, ↑*L. acidophilus* LA-5, ↑*Bifidobacteria*, ↑ *Lactobacilli*, ↓*Enterococci*			qPCR
13	Simon et al./2015/Multicenter/Non-industry [77]	No impact on microbiota, only ↑*L. reuterii*-probiotic bacteria used in the study		No changes of LPS and cytokines	NGS
14	Stenman et al./2016/Multicenter/Industry [79]		Probiotic: ↑propionic acid, butyric acid, and valeric acid,	Synbiotic: changes in zonulin and hsCRP were statistically significantly correlated with changes in trunk fat mass; ↑ LPS level, but no effect on inflammatory markers.	Limulus Amebocyte Lysate assay
15	Szulińska et al./2018a/Poland/Non-industry [81]			↓LPS level	kinetic assay
16	Tenore et al./2019/Italy/Non-industry [82]	↑ *Bifidobacterium* and ↑*Lactobacillus* population, and ↓ Bacteroides and ↓*Enterococcus* genera but predominantly in the control group followed by probiotic one and lactofermented control meal	↓TMAO blood level		culture-dependent
17	Trautvetter et al./2012/Germany/Industry [83]	↑*L. paracasei* and ↑ *Lactobacilli*			qPCR
18	Valentini et al./2015/Multicenter/Non-industry [85]	No significan influence on gut microbiota.			qPCR

qPCR—quantitative polymerase chain reaction, NGS—next generation sequencing, ELISA—enzyme-linked immunosorbent assays, FISH—fluorescent in situ hybridization, GC-MS—gas chromatography-mass spectrometry, LC-MS—liquid chromatography-mass spectrometry, DAPI—4′,6-diamidino-2-phenylindole, NMR—nuclear magnetic resonance, Tx—treatment, NEFA—non-estrified fatty acid, GC—gas chromatography, SCFAs—short chain fatty acids, EUB-positive—Eubacteria positive, HD—high dose, LPS—lipopolysaccharide, TMAO—trimethylamine-N-oxide, OTU—operational taxonomic unit, PICRUSt—Phylogenetic Investigation of Communities by Reconstruction of Unobserved States, KEGG—Kyoto Encyclopedia of Genes and Genomes, WHR—waist to hip ratio, hsCRP—high sensitivity C-reactive protein, ↑—elevated, ↓—lowered.

## Supplementary Materials

The following are available online at https://www.mdpi.com/2077-0383/9/6/1788/s1, Table S1. Baseline metabolic parameters in studies persons, Table S2. Smoking status and physical activity, Table S3. Trial discontinuations and adverse effects, Table S4. Risk of bias, Table S5. Effect sizes regarding metabolic outcomes, Table S6. Summary of the preventive outcome and changes in microbial composition and metabolites as well as anti-inflammatory effects and gut-barrier markers associated with probiotics administration; Figure S1. Funnel plot for endpoint BMI (SMD) in the present meta-analysis, Figure S2. Funnel plot for endpoint BMI (DM) in the present meta-analysis, Figure S3. Funnel plot for endpoint WC (SMD) in the present meta-analysis, Figure S4. Funnel plot for endpoint WC (DM) in the present meta-analysis, Figure S5. Funnel plot for WC change scores (SMD) in the present meta-analysis, Figure S6. Funnel plot for WC change scores (DM) in the present meta-analysis.
