# Supplementary material for: The Effect of Probiotics and Synbiotics on Risk Factors Associated with Cardiometabolic Diseases in Healthy People—A Systematic Review and Meta-Analysis with Meta-Regression of Randomized Controlled Trials
Source: J Clin Med. 2020 Jun 8;9(6):1788. doi: 10.3390/jcm9061788 (PMC7357153; doi:10.3390/jcm9061788)
Supplement: Supplementary file 1 [file jcm-09-01788-s001.zip › jcm-808048-PL-supplementary-for conversion/Supplementary tables S2-S6 and Supplementary figures.docx.docx]

**Table S1.** Excel file.

**Table S2.** Smoking status and physical activity.

| **No** | **Reference/Year/Country/Sponsorship** | **Tabacco Use, *n* (%)** | **Diet/Physical Activity** |
| --- | --- | --- | --- |
| 1 | Agerbaek et al./1995/Denmark/industry [29] | ND | Habitual/ND |
| 2 | Agerholm-Larsen et al./2000/Denmark/industry [30] | ND | Habitual/habitual |
| 3 | Ahn et al./2015/South Korea/Non-industry [31] | 11/12 | Data not shown |
| 4 | Ahn et al./2015a/South Korea/Non-industry [32] | 14/11.57 | ND/ND |
| 5 | Andrade et al./2009/Portugal/industry [33] | ND | Habitual with no fermented foods/ND |
| 6 | Bjerg et al./2015/Denamark/industry [34] | 0 | Regular with two test meals at the beginning and at the end/not high |
| 7 | Boesmans et al./2018/Belgium/Non-industry [35] | 2/6.67 | Regular with no strict dietary epproach (e.g. Veganism) and no pre- and probiotics/ND |
| 8 | Brahe et al./2015/Multicenter/Academic/industry [36] | ND | Habitual/ND |
| 9 | Bukowska et al./Poland/Non-industry [37] | 13/43.33 | Habitual/habitual |
| 10 | Chang et al./2011/South Korea/Non-industry/industry [38] | ND | Habitual/habitu al |
| 11 | Cox et al./2014/Multicenter/industry [39] | ND | ND with no probiotic foods/ND |
| 12 | de Roos et al./2017/Netherlands/Non-industry [40] | 7/11.67 | ND/ND |
| 13 | Fabian et al./2006/Austria/industry [41] | 0 | Habitual/ND |
| 14 | Gleeson et al./2012/United Kingdom/industry [42] | 0 | No fermented foods/regulated training schedule |
| 15 | Gohel et al./2016/India/Non-industry [43] | ND | ND/ND |
| 16 | Gomes et al./2017/Brazil/Non-industry [44] | ND | Regular with no probiotics and fermented diary foods/ND |
| 17 | Greany et al./2008/USA/Non-industry/industry [45] | ND | Habitual/habitual |
| 18 | Guillemard et al./2010/Multicenter/industry [46] | 375/37.5 | 2-week (14 days) period of diet control followed by 3 months (84 days) of study product consumption and a 1-month (28 days) follow-up phase with no product consumption |
| 19 | Hatakka et al./2008/Finland/industry [47] | ND | Habitual/habitual |
| 20 | Hibberd et al./2019/Multicenter/industry [48] | ND | Habitual/habitu al |
| 21 | Higashikawa et al./2016/Japan/Non-industry [49] | ND | Habitual/habitu al |
| 22a | Ibrahim et al./2018/Malaysia/Non-industry/industry [50] | 0/0 | ND/ND |
| 22b |  | 0/0 | ND/circuit training 3 times per week with 2 circuits of exercises from weeks 1–8 followed by 3 circuits of exercises from weeks 9–12. |
| 23 | Inoue et al./2018/Japan/Non-industry [51] | ND | Habitual/encouraged to perfromed exercises every day |
| 24 | Ito et al./2017/Japan/Non-industry [52] | 9/15.25 | Habitual with no extra fermented foods and antioxidative supplements/habitual |
| 25 | Ivey et al./2014/Australia/Non-industry/industry [53] | ND | Habitual/habitual |
| 26 | Ivey et al./2015/Australia/Non-industry/industry [54] | ND | Habitual/habitual |
| 27 | Jones et al./2016/Canada/industry [55] | ND | Habitual/ND |
| 28 | Kadooka et al./2010/Japan/nd [56] | ND | Habitual/habitual |
| 29 | Kadooka et al./2013/Japan/Non-industry [57] | ND | Habitual/habitual |
| 30 | Kawase et al./2000/Japan/Non-industry [58] | ND | ND/ND |
| 31a | Kim et al./2018/South Korea/Non-industry/Non-industry [59] | 12/20 | Habitual/habitual |
| 31b |  | 11/18.33 |  |
| 32 | Kim et al./2017/South Korea/Non-industry [60] | ND | Habitual/habitual |
| 33 | Klein et al./2008/Germany/Non-industry [61] | ND | Habitual but provided by staff/ND |
| 34 | Lambert et al./2017/Denmark/industry/Non-industry [62] | 9/15.25 | ND/avoidance of strenuous physical activity |
| 35 | Lee et al./2017/USA/Non-industry [63] | 0 | Habitual/habitual |
| 36 | Lin et al./1989/USA/industry [64] | ND | Habitual/exercise regularly every week |
| 37 | Macfarlane et al../2013/United Kingdom/Non-industry [65] | 3/7 | ND/ND |
| 38 | Madjd et al./2016/Multicenter/Non-industry [66] | ND | 500- to 1000-kcal energy deficit/60 min of moderate activity 5 d/wk |
| 39 | Mohammad Moradi et al./2015/Iran/non-industry [67] | ND | Habitual/habitual |
| 40 | Naruszewicz et al./2002/Poland/Non-industry [68] | 36/100 | Habitual/habitual |
| 41 | Nishiyama et al./2018/Japan/industry [69] | ND | Habitual with no probiotics/ND |
| 42 | Nova et al./2011/Spain/Non-industry [70] | ND | Habitual with no pre- and probiotics, fermented foods/ND |
| 43 | Ostan et al./2015/Multicenter/Non-industry [71] | ND | RISTOMED diet/ND |
| 44 | Osterberg et al./2015/USA/Non-industry [72] | ND | High-fat (55% fat) - high calorie (+1000kcal/day) diet/no vigorous physical activity |
| 45 | Rajkuma et al./2014/india/Non-industry [73] | ND | ND/ND |
| 46 | Sadrzadeh-Yeganeh et al./2010/Multicenter/industry [74] | ND | Habitual/habitual |
| 47 | Sanchez et al./2014/Multicenter/industry [75] | ND | Weight loss for 12 weeks followed by weight maintenance programme/no physical exercise 48 prepare to the testing |
| 48 | Savard et al./2011/Canada/industry [76] | ND | Habitual (of North America type)/habitual |
| 49 | Simon et al./2015/Multicenter/Non-industry [77] | ND | Habitual/ND |
| 50 | Simons et al./2006/Australia/Industry [78] | ND | Habitual/ND |
| 51 | Stenman et al./2016/Multicenter/industry [79] | ND | Habitual/habitual |
| 52 | Szulińska et al./2018/Poland/Non-industry [80] | ND | Habitual/habitual |
| 53 | Szulińska et al./2018a/Poland/Non-industry [81] | ND | Habitual/habitual |
| 54 | Tenore et al./2019/Italy/Non-industry [82] | 0 | Habitual/habitual |
| 55 | Trautvetter et al./2012/Germany/industry [83] | ND | Regular – provided by staff 3 days prior to the examination/ND |
| 56 | Usinger et al./2010/Denmark/Industry [84] | ND | Habitual/habitual |
| 57 | Valentini et al./2015/Multicenter/Non-industry [85] | ND | Ristomed diet/ND |
| 58 | Välimäki et al./2012/Finland/Non-industry [86] | ND | Habitual with no probiotics/ND |
| 59 | Venkataraman et al./2018/India/ND [87] | ND | Habitual/ND |
| 60 | Xiao et al./2003/Japan/industry/Non-industry [88] | ND | Habitual with no fermented foods/ND |
| 61 | Zarrati et al./2014/Iran/Non-industry [89] | ND | Low calorie diet |

ND- not determined.

**Table S3.** Trials discontinuations and adverse effects.

| **No** | **Reference/Year/Country/Sponsorship** | **Discontinuation: All cause** | | | | **Discontinuation: Adverse Events** | | | | |
| --- | --- | --- | --- | --- | --- | --- | --- | --- | --- | --- |
|  |  | **Probio Events** | **Probio *n*** | **PBO Events** | **PBO *n*** | **Probio Events** | **Probio *n*** | **PBO Events** | **PBO *n*** | **What is Adverse Event** |
| **1** | Agerbaek et al./1995/Denmark/industry [29] | 0 | 29 | 1 | 29 | 0 | 29 | 0 | 28 | NA |
| 2a | Agerholm-Larsen et al./2000/Denmark/industry [30] | 0 | 16 | 0 | 10 | 0 | 16 | 0 | 10 | NA |
| 2b |  | 1 | 15 | 0 | 10 | 1 | 15 | 0 | 10 | nausea and constipation |
| 2c |  | 0 | 16 | 0 | 10 | 0 | 16 | 1 | 10 | nausea and constipation |
| 3 | Ahn et al./2015/South Korea/Non-industry [31] | 0 | 46 | 0 | 46 | NA | NA | NA | NA | NA |
| 4 | Ahn et al./2015a/South Korea/Non-industry [32] | NA | NA | NA | NA | NA | NA | NA | NA | NA |
| 5a | Andrade et al./2009/Portugal/industry [33] | 0 | 19 | 0 | 15 | 0 | 19 | 0 | 15 | NA |
| 5b |  | 0 | 15 | 0 | 19 | 0 | 15 | 0 | 19 | NA |
| 6 | Bjerg et al./2015/Denamark/industry [34] | 6 * | nd | 6 | nd | 0 | NA | 0 | NA | NA |
| 7 | Boesmans et al./2018/Belgium/Non-industry [35] | 2 ** | 30 | 2 | 30 | 0 | 30 | 0 | 30 | NA |
| 8 | Brahe et al./2015/Multicenter/Academic/industry [36] | 1 | 19 | 4 | 20 | 0 | 19 | 0 | 20 | NA |
| 9 | Bukowska et al./Poland/Non-industry [37] | ND | ND | ND | ND | ND | ND | ND | ND | ND |
| 10 | Chang et al./2011/South Korea/Non-industry/industry [38] | 2 ^ | 103 | 2^ | 103 | 0 | 53 | 0 | 48 | NA |
| 11a | Cox et al./2014/Multicenter/industry [39] | 0 | 39 | 0 | 45 | 0 | 39 | 0 | 45 | NA |
| 11b |  | 0 | 45 | 0 | 45 | 0 | 45 | 0 | 45 | NA |
| 12 | de Roos et al./2017/Netherlands/Non-industry [40] | 0 | 31 | 3 | 32 | ND | 31 | ND | 32 | NA |
| 13 | Fabian et al./2006/Austria/industry [41] | 1^ | 17/18 | 1^ | 16/17 | ND | ND | ND | ND | NA |
| 14 | Gleeson et al./2012/United Kingdom/industry [42] | 1 *** | 33 | 1 *** | 33 | ND | ND | ND | ND | metabolic anomalies but no data whether these were coaused by probiotics |
| 15 | Gohel et al./2016/India/Non-industry [43] | 5 | 39 | 12 | 39 | ND | ND | ND | ND | NA |
| 16 | Gomes et al./2017/Brazil/Non-industry [44] | 9 | 30 | 8 | 30 | 0 | 21 | 0 | 22 | NA |
| 17 | Greany et al./2008/USA/Non-industry/industry [45] | 9^ | ND | 9^ | ND | 0 | 37 | 0 | 18 | NA |
| 18 | Guillemard et al./2010/Multicenter/industry[46] | 22 | 500 | 16 | 500 | 7 | 500 | 5 | 500 | not specified: "unacceptable adverse events" |
| 19 | Hatakka et al./2008/Finland/industry [47] | 0 | 38 | 0 | 38 | 0 | 38 | 0 | 38 | NA |
| 20a | Hibberd et al./2019/Multicenter/industry [48] | ND | ND | ND | ND | ND | ND | ND | ND | NA |
| 20b |  | ND | ND | ND | ND | ND | ND | ND | ND | NA |
| 21a | Higashikawa et al./2016/Japan/Non-industry [49] | 2 | 21 | 0 | 20 | 1 | 21 | 0 | 20 | constipation |
| 21b |  | 2 | 21 | 0 | 20 | 1 | 21 | 0 | 20 | hives |
| 22a | Ibrahim et al./2018/Malaysia/Non-industry/industry [50] | 2 | 12 | 2 | 12 | NA | NA | NA | NA | NA |
| 22b |  | 3 | 12 | 0 | 12 | NA | NA | NA | NA | NA |
| 23 | Inoue et al./2018/Japan/Non-industry [51] | 0 | 20 | 1 | 19 | 0 | 20 | 0 | 19 | na |
| 24 | Ito et al./2017/Japan/Non-industry [52] | 1 | 30 | 0 | 30 | 0 | 30 | 0 | 30 | NA |
| 25a | Ivey et al./2014/Australia/Non-industry/industry [53] | 1 | 40 | 3 | 37 | 0 | 40 | 0 | 37 | NA |
| 25b |  | 1 | 39 | 0 | 40 | 0 | 39 | 0 | 40 | NA |
| 26a | Ivey et al./2015/Australia/Non-industry/industry [54] | 1 | 40 | 3 | 37 | 0 | 40 | 0 | 37 | NA |
| 26b |  | 1 | 39 | 0 | 40 | 0 | 39 | 0 | 40 | NA |
| 27 | Jones et al./2016/Canada/industry [55] | 1 | 67 | 3 | 64 | NA | NA | NA | NA | NA |
| 28 | Kadooka et al./2010/Japan/nd 56] | 0 | 43 | 0 | 44 | 0 | 43 | 0 | 44 | NA |
| 29a | Kadooka et al./2013/Japan/Non-industry [57] | 0 | 69 | 0 | 70 | 0 | 69 | 0 | 70 | NA |
| 29b |  | 0 | 71 | 0 | 70 | 0 | 71 | 0 | 70 | NA |
| 30 | Kawase et al./2000/Japan/Non-industry [58] | NA | NA | NA | NA | NA | NA | NA | NA | NA |
| 31a | Kim et al./2018/South Korea/Non-industry/Non-industry [59] | 4 | 30 | 5 | 30 | 0 | 30 | 0 | 30 | NA |
| 31b |  | 4 | 30 | 5 | 30 | 0 | 30 | 0 | 30 | NA |
| 32 | Kim et al./2017/South Korea/Non-industry [60] | NA | 32 | NA | 34 | NA | NA | NA | NA | NA |
| 33 | Klein et al./2008/Germany/Non-industry [61] | 0 | 26 | 0 | 26 | 0 | 26 | 0 | 26 | NA |
| 34 | Lambert et al./2017/Denmark/industry/Non-industry [62] | 1 | 30 | 2 | 29 | 0 | 30 | 0 | 29 | NA |
| 35a | Lee et al./2017/USA/Non-industry [63] | 6 | 30 | 6 | 30 | 1 | 30 | 1 | 30 | discomfort |
| 35b |  | 6 | 30 | 6 | 30 | 1 | 30 | 1 | 30 |  |
| 35c |  | 6 | 30 | 6 | 30 | 1 | 30 | 1 | 30 |  |
| 36a | Lin et al./1989/USA/industry [64] | 33 | 157 | 27 | 177 | 33 | 157 | 27 | 177 | constipatio, flatulence, diarrhea, stomach upset |
| 36b |  | 10 | 177 | 11 | 157 | 10 | 177 | 11 | 157 | constipation, flatulence, diarrhea, stomach upset |
| 37 | Macfarlane et al../2013/United Kingdom/Non-industry [65] | 1 | 23 | 3 | 20 | 0 | 23 | 0 | 20 | NA |
| 38 | Madjd et al./2016/Multicenter/Non-industry [66] | 5 | 44 | 3 | 45 | 0 | 44 | 0 | 45 | NA |
| 39a | Mohammad Moradi et al./2015/Iran/non-industry [67] | 0 | 60 | 0 | 60 | 0 | 60 | 0 | 60 | NA |
| 39b |  | 0 | 60 | 0 | 60 | 0 | 60 | 0 | 60 | NA |
| 40 | Naruszewicz et al./2002/Poland/Non-industry [68] | 0 | 18 | 0 | 18 | 0 | 18 | 0 | 18 | NA |
| 41 | Nishiyama et al./2018/Japan/industry [69] | 3^ | 76 | 3^ | 76 | 0 | 76 | 0 | 76 | NA |
| 42 | Nova et al./2011/Spain/Non-industry [70] | 1 | 18 | 0 | 18 | 0 | 18 | 0 | 18 | NA |
| 43 | Ostan et al./2015/Multicenter/Non-industry [71] | 4 | 31 | 3 | 31 | 3 | 31 | 2 | 31 | dental infection, chest tightness, sleep disturbance, stomach pain, inflammatory status |
| 44 | Osterberg et al./2015/USA/Non-industry [72] | 0 | 9 | 0 | 11 | 0 | 9 | 0 | 11 | NA |
| 45a | Rajkuma et al./2014/india/Non-industry [73] | ND | ND | ND | ND | ND | ND | ND | ND | NA |
| 45b |  | ND | ND | ND | ND | ND | ND | ND | ND | NA |
| 46 | Sadrzadeh-Yeganeh et al./2010/Multicenter/industry [74] | 1 | 30 | 1 | 30 | 0 | 30 | 0 | 30 | NA |
| 47 | Sanchez et al./2014/Multicenter/industry [75] | 17 | 62 | 15 | 63 | 0 | 62 | 0 | 63 | NA |
| 48a | Savard et al./2011/Canada/industry [76] | 1 | 20 | 1 | 20 | ND | 20 | ND | 20 | flatulance, abdominal bloating, cramps, reflux, nausea, constipation, diarrhea, belching, headaches, urinary frequence, stool frequence, apetite and quantity of ingested food |
| 48b |  | 1 | 18 | 1 | 20 | ND | 18 | ND | 20 |  |
| 49 | Simon et al./2015/Multicenter/Non-industry [77] | 0 | 11 | 0 | 10 | 0 | 11 | 0 | 10 | none of the participants reported any adverse effects including gastrointestinal disorders |
| 50 | Simons et al./2006/Australia/Industry [78] | 2 | 25 | 0 | 21 | 1 | 25 | 0 | 21 | bowel discomfort |
| 51a | Stenman et al./2016/Multicenter/industry [79] | 23 | 48 | 20 | 56 | ND | 48 | ND | 56 | 199 adverse events in 244 persons: nasopharyngitis, diarrhea, headache, flatulance, influenza |
| 51b |  | 15 | 52 | 20 | 57 | ND | 52 | ND | 57 |  |
| 52a | Szulińska et al./2018/Poland/Non-industry [80] | 4 | 27 | 3 | 27 | 0 | 27 | 0 | 27 | no adverse events |
| 52b |  | 3 | 27 | 3 | 27 | 1 | 27 | 0 | 27 | cardiac events |
| 53a | Szulińska et al./2018a/Poland/Non-industry [81] | 4 | 27 | 3 | 27 | 0 | 27 | 0 | 27 | no adverse events |
| 53b |  | 3 | 27 | 3 | 27 | 1 | 27 | 0 | 27 | cardiac events |
| 54a | Tenore et al./2019/Italy/Non-industry [82] | 6 | 27 | 6 | 27 | ND | ND | ND | ND | NA |
| 54b |  | 6 | 26 | 6 | 27 | ND | ND | ND | ND | NA |
| 55 | Trautvetter et al./2012/Germany/industry [83] | 2^ | 32 | 2^ | 32 | ND | ND | ND | ND | NA |
| 56a | Usinger et al./2010/Denmark/Industry [84] | 2 | 32 | 0 | 15 | ND | ND | ND | ND | 1 person had grade II hypertension on the 24hBP, 1 had atrial fibrillation at baseline and 1 had increased plasma potassium at baseline |
| 56b |  | 3 | 32 | 0 | 15 | ND | ND | ND | ND | NA |
| 57 | Valentini et al./2015/Multicenter/Non-industry -85] | 4 | 35 | 3 | 34 | 2 | 35 | 1 | 34 | dental infection (antibiotics) or breast oppression drug for more then 3 days, sleep disturbance, stomach pain, inflammatory state (antiinflammatory drug |
| 58 | Välimäki et al./2012/Finland/Non-industry [86] | 22^ | 61 | 22^ | 58 | ND | ND | ND | ND | Of the 22 subjects who dropped out of the study, 17 could not take part in the marathon and training because of injury or serious illness and 5 refused to continue |
| 59 | Venkataraman et al./2018/India/ND [87] | ND | ND | ND | ND | ND | ND | ND | ND | NA |
| 60 | Xiao et al./2003/Japan/industry/Non-industry [88] | 0 | 16 | 0 | 16 | 0 | 16 | 0 | 16 | NA |
| 61a | Zarrati et al./2014/Iran/Non-industry [89] | ND | 25 | ND | 25 | ND | 25 | ND | 25 | NA |
| 61b |  | ND | 26 | ND | 26 | ND | 26 | ND | 26 | NA |

ND- not determined, NA – not applicable, * 6 out of 70 were excluded but the group was not specified and these were not adverse events, ** 2 out from 1st phase (Probiotic – Placebo), ^- the group is not specified (discontinuations are doubled in two groups), *** 1 excluded but the phase was not specified (discontinuations are doubled in two phases).

**Table S4.** Risk of bias.

| **No.** | **Reference/Year/Country/Sponsorship** | **Random Sequence Generation (Selection Bias)** | **Allocation Concealment (Selection Bias)** | **Blinding of Participants and Personnel (Performance Bias)** | **Blinding of Outcome Assessment (Detection Bias)** | **Incomplete Outcome Data Addressed (Attrition Bias)** | **Selective Reporting (Reporting Bias)** | **Other Bias** | **No. of Low Assesments** |
| --- | --- | --- | --- | --- | --- | --- | --- | --- | --- |
| 1 | Agerbaek et al./1995/Denmark/industry [29] | ? | ? | ? | ? | L | L | ? | 2 |
| 2 | Agerholm-Larsen et al./2000/Denmark/industry [30] | ? | ? | ? | ? | L | L | ? | 2 |
| 3 | Ahn et al./2015/South Korea/Non-industry [31] | L | L | ? | ? | H | ? | ? | 2 |
| 4 | Ahn et al./2015a/South Korea/Non-industry [32] | L | L | ? | ? | H | ? | ? | 2 |
| 5 | Andrade et al./2009/Portugal/industry [33] | ? | ? | ? | ? | ? | H | ? | 0 |
| 6 | Bjerg et al./2015/Denamark/industry [34] | L | L | L | H | H | H | ? | 3 |
| 7 | Boesmans et al./2018/Belgium/Non-industry [35] | L | L | L | L | L | L | L | 7 |
| 8 | Brahe et al./2015/Multicenter/Academic/industry [36] | H | H | H | H | L | L | ? | 3 |
| 9 | Bukowska et al./Poland/Non-industry [37] | ? | ? | ? | ? | L | L | ? | 2 |
| 10 | Chang et al./2011/South Korea/Non-industry/industry [38] | ? | ? | ? | ? | L | L | ? | 2 |
| 11 | Cox et al./2014/Multicenter/industry [39] | L | L | ? | ? | ? | ? | ? | 2 |
| 12 | de Roos et al./2017/Netherlands/Non-industry [40] | L | L | ? | ? | ? | L | L | 4 |
| 13 | Fabian et al./2006/Austria/industry [41] | ? | ? | ? | ? | ? | ? | ? | 0 |
| 14 | Gleeson et al./2012/United Kingdom/industry [42] | ? | ? | ? | ? | H | L | ? | 1 |
| 15 | Gohel et al./2016/India/Non-industry [43] | L | L | ? | ? | ? | L | ? | 3 |
| 16 | Gomes et al./2017/Brazil/Non-industry [44] | L | L | L | ? | L | L | ? | 5 |
| 17 | Greany et al./2008/USA/Non-industry/industry [45] | ? | ? | H | ? | L | L | ? | 2 |
| 18 | Guillemard et al./2010/Multicenter/industry [46] | L | L | ? | L | H | ? | ? | 3 |
| 19 | Hatakka et al./2008/Finland/industry [47] | ? | ? | ? | ? | L | L | ? | 2 |
| 20 | Hibberd et al./2019/Multicenter/industry [48] | ? | ? | ? | ? | H | L | ? | 1 |
| 21 | Higashikawa et al./2016/Japan/Non-industry [49] | L | L | L | L | L | L | ? | 6 |
| 22 | Ibrahim et al./2018/Malaysia/Non-industry/industry [50] | ? | ? | ? | ? | L | ? | ? | 1 |
| 23 | Inoue et al./2018/Japan/Non-industry [51] | ? | L | L | ? | L | L | ? | 4 |
| 24 | Ito et al./2017/Japan/Non-industry [52] | L | L | L | ? | L | L | ? | 5 |
| 25 | Ivey et al./2014/Australia/Non-industry/industry [53] | L | L | L | ? | H | L | ? | 4 |
| 26 | Ivey et al./2015/Australia/Non-industry/industry [54] | L | L | L | ? | H | L | ? | 4 |
| 27 | Jones et al./2016/Canada/industry [55] | L | L | L | ? | L | L | ? | 5 |
| 28 | Kadooka et al./2010/Japan/nd [56] | ? | ? | ? | ? | L | L | ? | 2 |
| 29 | Kadooka et al./2013/Japan/Non-industry [57] | ? | ? | ? | ? | L | L | ? | 2 |
| 30 | Kawase et al./2000/Japan/Non-industry [58] | ? | H | H | H | L | L | H | 2 |
| 31 | Kim et al./2018/South Korea/Non-industry/Non-industry [59] | L | L | L | ? | L | L | ? | 5 |
| 32 | Kim et al./2017/South Korea/Non-industry [60] | L | L | L | ? | L | L | ? | 5 |
| 33 | Klein et al./2008/Germany/Non-industry [61] | ? | L | L | ? | ? | L | ? | 3 |
| 34 | Lambert et al./2017/Denmark/industry/Non-industry [62] | ? | L | L | ? | L | L | ? | 4 |
| 35 | Lee et al./2017/USA/Non-industry [63] | ? | L | H | ? | L | L | ? | 3 |
| 36 | Lin et al./1989/USA/industry [64] | L | L | L | ? | L | ? | ? | 4 |
| 37 | Macfarlane et al../2013/United Kingdom/Non-industry [65] | L | L | L | ? | L | L | ? | 5 |
| 38 | Madjd et al./2016/Multicenter/Non-industry [66] | ? | L | H | H | L | L | ? | 3 |
| 39 | Mohammad Moradi et al./2015/Iran/non-industry [67] | L | L | L | ? | L | L | ? | 5 |
| 40 | Naruszewicz et al./2002/Poland/Non-industry [68] | ? | L | ? | ? | L | L | ? | 3 |
| 41 | Nishiyama et al./2018/Japan/industry [69] | ? | H | L | ? | L | ? | ? | 2 |
| 42 | Nova et al./2011/Spain/Non-industry [70] | ? | L | L | ? | L | ? | ? | 3 |
| 43 | Ostan et al./2015/Multicenter/Non-industry [71] | L | L | H | ? | L | ? | ? | 3 |
| 44 | Osterberg et al./2015/USA/Non-industry [72] | ? | L | L | ? | L | ? | ? | 3 |
| 45 | Rajkuma et al./2014/india/Non-industry [73] | L | L | L | L | L | L | ? | 6 |
| 46 | Sadrzadeh-Yeganeh et al./2010/Multicenter/industry [74] | ? | L | ? | ? | L | ? | ? | 2 |
| 47 | Sanchez et al./2014/Multicenter/industry [75] | ? | L | L | ? | L | L | ? | 4 |
| 48 | Savard et al./2011/Canada/industry [76] | ? | L | L | ? | L | ? | ? | 3 |
| 49 | Simon et al./2015/Multicenter/Non-industry [77] | ? | L | L | ? | L | L | ? | 4 |
| 50 | Simons et al./2006/Australia/Industry [78] | ? | L | L | ? | L | ? | ? | 3 |
| 51 | Stenman et al./2016/Multicenter/industry [79] | L | L | L | ? | L | ? | ? | 4 |
| 52 | Szulińska et al./2018/Poland/Non-industry [80] | L | L | L | ? | L | L | ? | 5 |
| 53 | Szulińska et al./2018a/Poland/Non-industry [81] | L | L | L | ? | L | L | ? | 5 |
| 54 | Tenore et al./2019/Italy/Non-industry [82] | L | L | L | L | L | L | ? | 6 |
| 55 | Trautvetter et al./2012/Germany/industry [83] | ? | L | L | ? | L | ? | ? | 3 |
| 56 | Usinger et al./2010/Denmark/Industry [84] | ? | L | L | L | L | L | ? | 5 |
| 57 | Valentini et al./2015/Multicenter/Non-industry [85] | ? | L | L | ? | L | L | ? | 4 |
| 58 | Välimäki et al./2012/Finland/Non-industry [86] | ? | L | L | ? | L | L | ? | 4 |
| 59 | Venkataraman et al./2018/India/ND [87] | ? | L | H | ? | L | ? | ? | 2 |
| 60 | Xiao et al./2003/Japan/industry/Non-industry [88] | ? | L | H | ? | L | ? | ? | 2 |
| 61 | Zarrati et al./2014/Iran/Non-industry [89] | ? | L | L | ? | L | ? | ? | 3 |

L-low risk of bias, H - high risk of bias, ? - unclear risk of bias.

**Table S5.** Excel file.

**Table S6.** Summary of the preventive outcome and changes in microbial composition and metabolites as well as anti-inflammatory effects and gut barrier markers associated with probiotics administration.

| **Reference/Year/Country/Sponsorship** | **Preventive Outcome** | **Microbiota Composition Changes** | **Microbial Metabolites Changes** | **Anti-Inflammatory Activity / Gut Barrier Markers** | **Comments** |
| --- | --- | --- | --- | --- | --- |
| Boesmans et al./2018/Belgium/Non-industry [35] | NO | NO | NO | NA / NO | - |
| Brahe et al./2015/Multicenter/Academic/Industry [36] | NO | YES | NO | NO / NO | - |
| de Roos et al./2017/Netherlands/Non-industry [40] | NA | NA | NA | NO / NO | The following markers were measured: inflammation: IL-6, IL-10, TNFα and CRP / gut barrier: zonulin concentration and lactulose-mannitol test |
| Hibberd et al. /2019/Multicenter/Industry [48] | NO | YES | YES | NA / NA | Positive and negative correlation of different bacteria to metabolic parameters |
| Jones et al./2016/Canada/Industry [55] | YES | NA | NA | NO / NO | - |
| Klein et al./2008/Germany/Non-industry [61] | YES | YES | NO | ? / NO | Increase of a marker of unspecific cellular response |
| Lee et al./2017/USA/Non-industry [63] | NO | NA | ? | NO / NO | Inconsistent results, faecal acetate change in on probiotic and control group in cross-over study |
| Macfarlane et al./2013/United Kingdom/Non-industry [65] | NO | YES | YES | YES / YES | - |
| Osterberg et al./2015/USA/Non-industry [72] | YES | YES | NA | NO / NO | - |
| Rajkumar et al./2014/India/Non-industry [73] | YES | YES | NA | YES / YES | - |
| Sanchez et al./2014/Multicenter/Industry [75] | YES | YES | NO | NO / NO | Microbiota changes observed only in women |
| Savard et al./2011/Canada/Industry [76] | YES | YES | NA | NA / NA | - |
| Simon et al./2015/Multicenter/Non-industry [77] | YES | NO | NA | NO / NO | - |
| Stenman et al./2016/Multicenter/Industry [79] | YES | NA | YES | ? / ? | Inconsistent data: no influence on HsCRP and zonulin, but changes in zonulin and hsCRP in symbiotic group were correlated with changes in trunk fat mass; ↑ LPS level but no effect on inflammatory markers. |
| Szulińska et al. /2018a/Poland/Non-industry [81] | YES | NA | NA | NO / YES | - |
| Tenore et al./2019/Italy/Non-industry [82] | YES | YES | YES | NA / NA | ↓TMAO blood level |
| Trautvetter et al./2012/Germany/Industry [83] | NO | YES | NA | NA /NA | - |
| Valentini et al./2015/Multicenter/ [85]  Non-industry | YES | NO | NA | NA | - |

NO-not observed, YES-observed, NA- not analysed ? – results are not clear, IL – interleukin, TNFα - tumor necrosis factorα, CRP – c reactive protein, Tregs - regulatory T cell, SCFA-short chain fatty acid, CFA-cellular fatty acid. TMAO—trimethylamine-N-oxide, HsCRP—high sensitivity C-reactive protein.


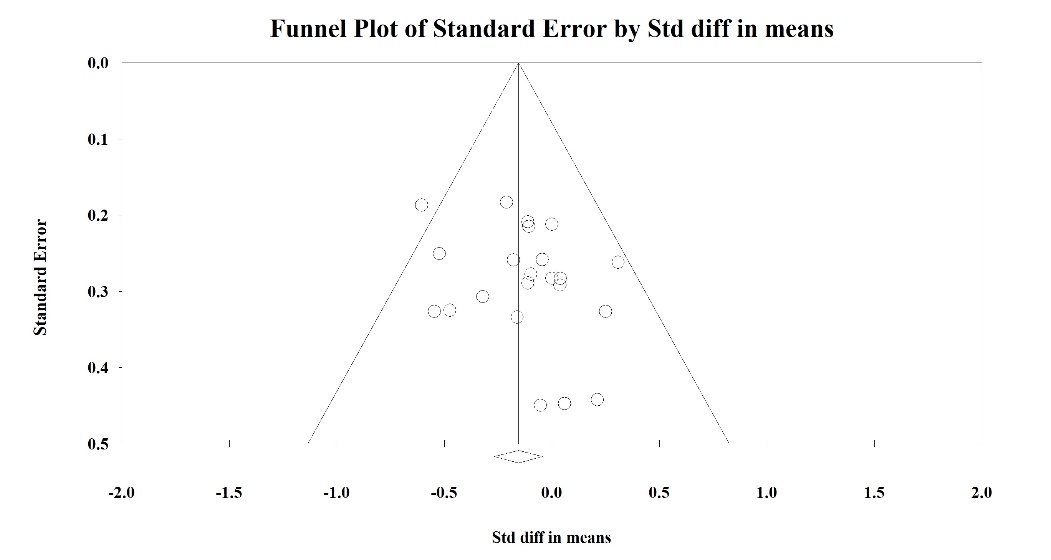


**Figure S1.** Funnel plot for endpoint BMI (SMD) in present meta-analysis. BMI—body mass index, SMD—standardized mean difference.


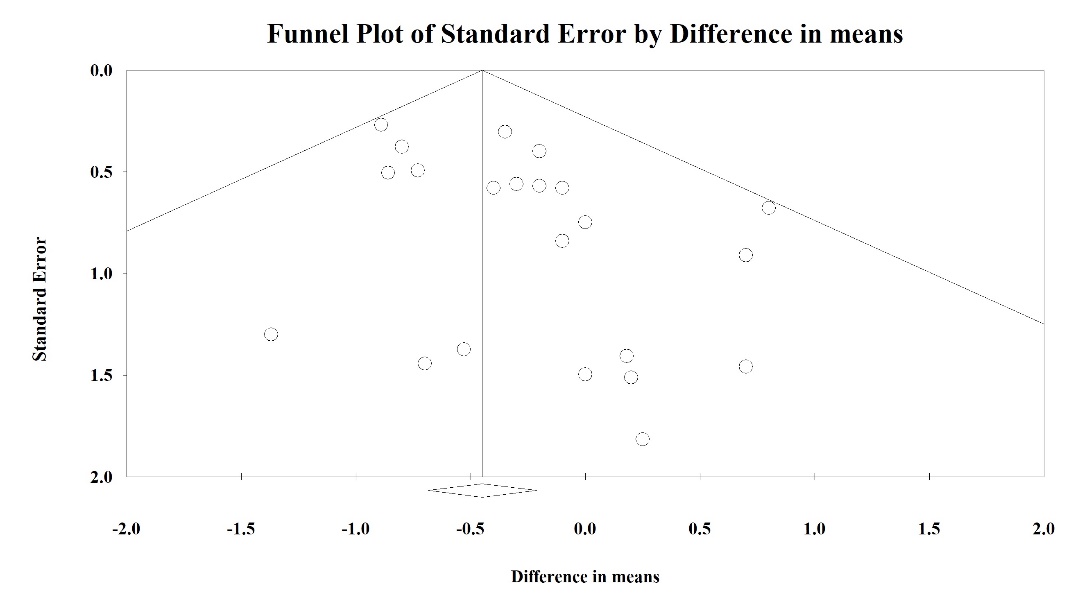


**Figure S2.** Funnel plot for endpoint BMI (DM) in present meta-analysis. DM—difference in means.


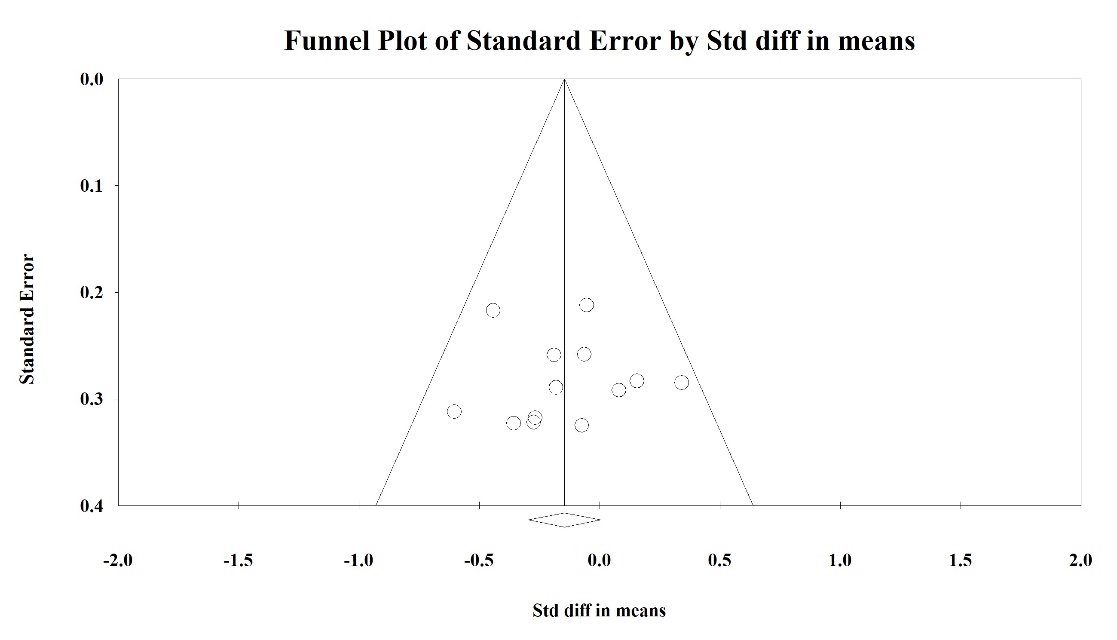


**Figure S3.** Funnel plot for endpoint WC (SMD) in present meta-analysis. WC—waist circumference.


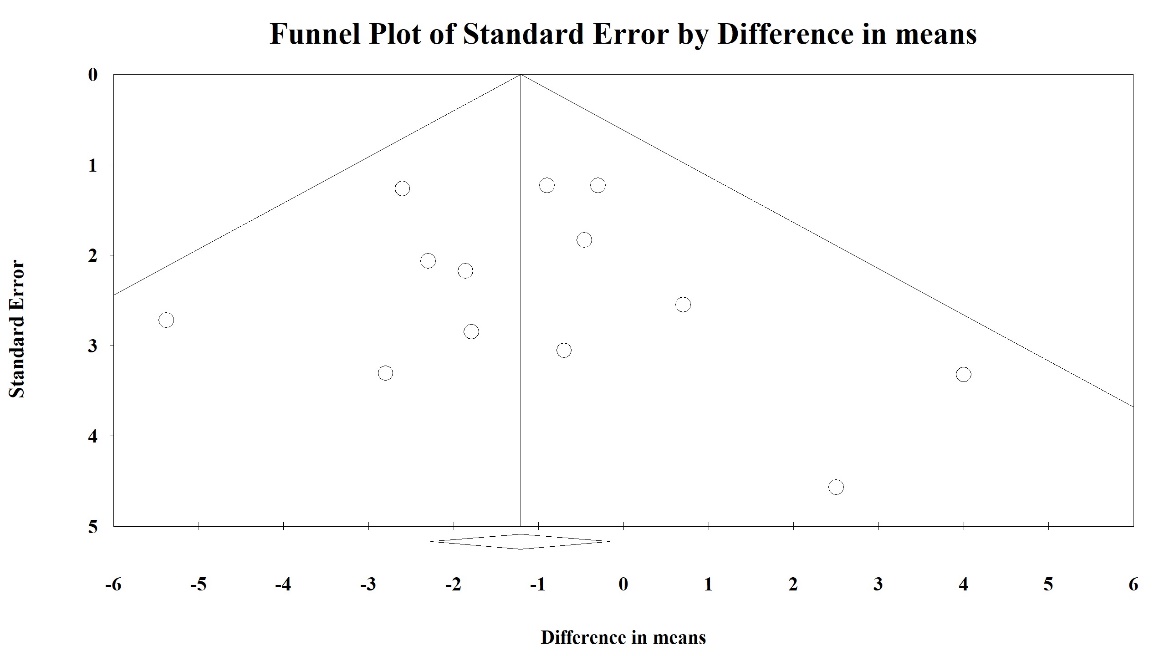


**Figure S4.** Funnel plot for endpoint WC (DM) in present meta-analysis.


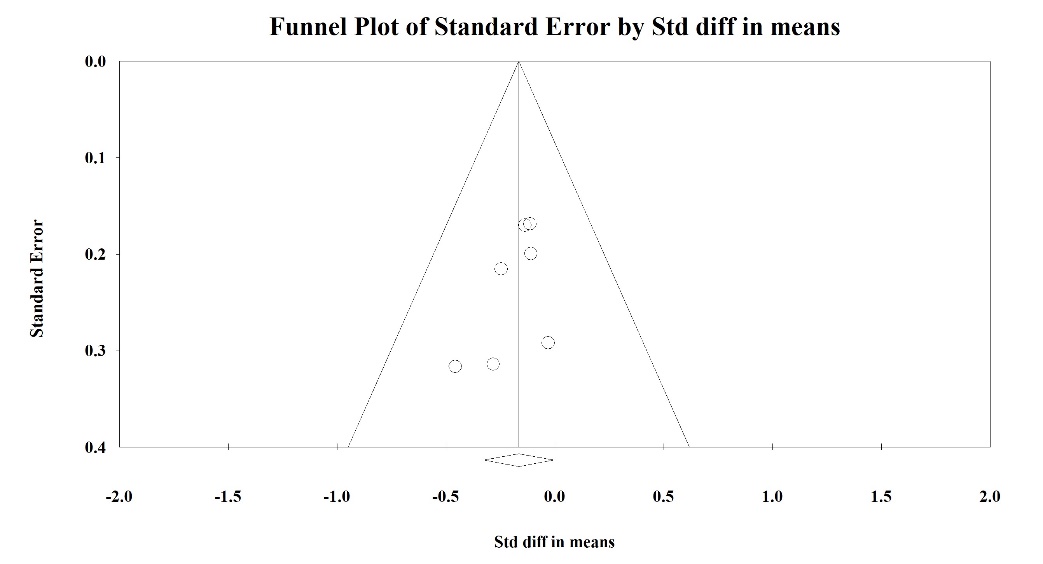


**Figure S5.** Funnel plot for WC change scores (SMD) in present meta-analysis.


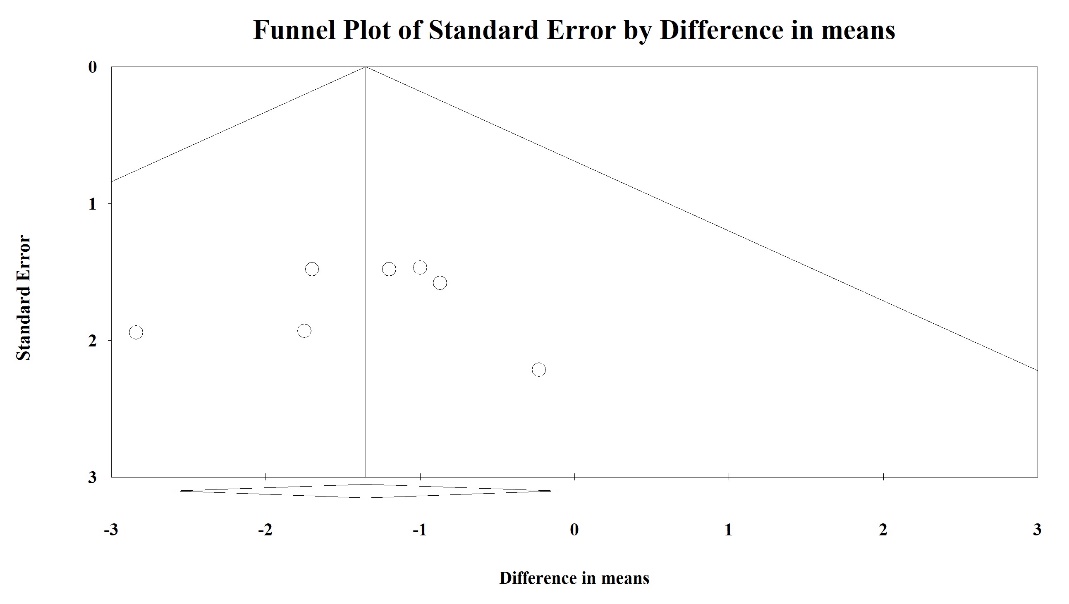


**Figure S6.** Funnel plot for WC change scores (DM) in present meta-analysis.
